# Effects of the multi‐kinase inhibitor midostaurin in combination with chemotherapy in models of acute myeloid leukaemia

**DOI:** 10.1111/jcmm.14927

**Published:** 2020-01-22

**Authors:** Ellen Weisberg, Chengcheng Meng, Abigail E. Case, Hong L. Tiv, Prafulla C. Gokhale, Sara J. Buhrlage, Jing Yang, Xiaoxi Liu, Jinhua Wang, Nathanael Gray, Sophia Adamia, Martin Sattler, Richard Stone, James D. Griffin

**Affiliations:** ^1^ Department of Medical Oncology Dana‐Farber Cancer Institute Boston MA USA; ^2^ Department of Medicine Harvard Medical School Boston MA USA; ^3^ Experimental Therapeutic Core Dana‐Farber Cancer Institute Harvard Medical School Boston MA USA; ^4^ Department of Cancer Biology Dana‐Farber Cancer Institute Harvard Medical School Boston MA USA; ^5^ Department of Biological Chemistry and Molecular Pharmacology Harvard Medical School Boston MA USA

**Keywords:** acute myeloid leukaemia, crenolanib, gilteritinib, midostaurin, non‐mutant FLT3, quizartinib, sorafenib, SYK, synergy

## Abstract

Recently, several targeted agents have been developed for specific subsets of patients with acute myeloid leukaemia (AML), including midostaurin, the first FDA‐approved FLT3 inhibitor for newly diagnosed patients with FLT3 mutations. However, in the initial Phase I/II clinical trials, some patients without FLT3 mutations had transient responses to midostaurin, suggesting that this multi‐targeted kinase inhibitor might benefit AML patients more broadly. Here, we demonstrate submicromolar efficacy of midostaurin in vitro and efficacy in vivo against wild‐type (wt) FLT3‐expressing AML cell lines and primary cells, and we compare its effectiveness with that of other FLT3 inhibitors currently in clinical trials. Midostaurin was found to synergize with standard chemotherapeutic drugs and some targeted agents against AML cells without mutations in FLT3. The mechanism may involve, in part, the unique kinase profile of midostaurin that includes proteins implicated in AML transformation, such as SYK or KIT, or inhibition of ERK pathway or proviability signalling. Our findings support further investigation of midostaurin as a chemosensitizing agent in AML patients without FLT3 mutations.

## 1 INTRODUCTION

Mutations in the tyrosine kinase (TK) FLT3 represent one of the most prevalent and one of the few clinically validated targets in acute myeloid leukaemia (AML). Two major classes of FLT3 mutations exist in AML: (a) internal tandem duplication (ITD) in the juxtamembrane domain, detected in around 23% of AML cases, and (b) point mutations in the tyrosine kinase domain (TKD), identified in 7% of AML patients.^1^ Mutations also occur in other members of the class III receptor tyrosine kinase family, including c‐KIT, which can be expressed as ITDs with an overall incidence of 17%,^2^ and platelet‐derived growth factor receptors α and β (PDGFRA and PDGFRB), which are more rare.^3^, ^4^

Midostaurin (PKC412; Rydapt; Novartis Pharma AG) was the first FLT3 inhibitor^5^ approved for AML (RATIFY [CALGB 10603]).^6^, ^7^ Although originally pursued for mutant FLT3‐positive AML, clinical results show it is also efficacious against wild‐type (wt) FLT3‐expressing AML.^8^, ^9^ This clinical benefit may be due to various factors, including overexpression of wt FLT3 that occurs in 70%‐100% of AML cases,^10^, ^11^ or the broad spectrum of targets of midostaurin and its metabolites that may play a role in their capacity to kill leukaemia cells not driven by oncogenic FLT3, including kinases implicated in cellular transformation.^12^ In addition to mutant FLT3, midostaurin has other cell surface kinase receptors as targets, including VEGFR‐2, PDGFR‐α and PDGFR‐β, and c‐KIT; these proteins contribute to AML cell growth and survival.^12^ Numerous genetic mutations have recently been identified as correlating with clinical response to midostaurin, including mutations in *BCOR*, *JAK2*, *RUNX1*, *SRSF2*, *USAF1* and *PTPN11*, whereas lack of clinical response to midostaurin correlates with mutations in *IDH2*, *NPM1*, *WT1* and *GATA2*.^13^

Other clinically investigated FLT3 inhibitors are classified as either first‐generation inhibitors (relatively non‐specific for FLT3) or second‐generation inhibitors (more potent and selective for FLT3). Among these are the multi‐targeted, first‐generation FLT3 inhibitor sorafenib (Nexavar; co‐developed and co‐marketed by Bayer and Onyx Pharmaceuticals),^14^ which in addition to FLT3 also has VEGFR‐1‐3, PDGFR‐β, RET and c‐KIT as targets. Second‐generation FLT3 inhibitors include quizartinib (AC220; Daiichi Sankyo),^15^, ^16^ a highly selective inhibitor of FLT3‐ITD that does not inhibit FLT3‐TKD (D835 or F691) in patients,^17^ crenolanib besylate (CP‐868596; AROG Pharmaceuticals, LLC),^18^ which has FLT3‐ITD and FLT3‐TKD and PDGFR as targets, and the FLT3 and AXL inhibitor, gilteritinib (ASP2215, XOSPATA; Astellas Pharma US, Inc),^19^, ^20^ which was FDA‐approved for patients with relapsed or refractory AML with an identified FLT3 mutation, based on results of the ADMIRAL trial (NCT02421939).

As midostaurin has not been investigated largely in wt FLT3‐expressing AML pre‐clinically, we sought to explore its activity in this context, alone and in combination, to better understand the observed clinical efficacy and inform best combinations for future clinical investigation. It has been shown that for AML patients to achieve maximum clinical benefit, it is imperative that midostaurin be administered in combination with other anticancer agents.^6^, ^8^ Here, we investigate the effects of midostaurin alone against AML characterized as expressing either wt or mutant FLT3, as well as in combination with standard chemotherapy and targeted inhibitors. Our results suggest that midostaurin, with its unique kinase profile and broad‐spectrum activity, could potentially be used clinically as a chemosensitizing agent for AML patients expressing either mutated or non‐mutated FLT3.

## 2 MATERIALS AND METHODS

### 2.1 Cell lines

Ba/F3 (interleukin [IL]‐3‐dependent murine pro‐B) cells engineered to express SYK‐TEL, TEL‐SYK or FLT3‐ITD, provided by Dr Kimberly Stegmaier (Dana‐Farber Cancer Institute, MA), have been previously described.^21^, ^22^ The human AML‐derived, FLT3‐ITD–expressing line, MOLM14,^23^ was provided to us by Dr Scott Armstrong (Dana‐Farber Cancer Institute, MA). Kasumi‐1‐luc+, NB4‐luc+ and SKNO‐1‐luc+ cells were gifts from Dr Andrew Kung (Memorial Sloan Kettering Cancer Center, NY). HEL, HL60 and K052 cell lines were purchased from ATCC (Manassas, VA). SKM‐1, NOMO‐1, OCI‐AML2 and OCI‐AML3 cell lines were obtained from Dr Gary Gilliland (Fred Hutchinson Cancer Research Center, WA).

More details are provided in Appendix S1.

### 2.2 Chemical compounds and biologic reagents

Details are provided in the Appendix S1.

### 2.3 Cell proliferation studies and apoptosis studies

Details are provided in the Appendix S1.

### 2.4 Immunoblotting

Protein lysate preparation and immunoblotting were carried out as previously described.^5^

### 2.5 Antibodies

Antibodies purchased from Cell Signaling Technology were used at a dilution of 1:1000 and include phospho‐S6 ribosomal protein (S235/236) (D57.2.2E) XP (R) (rabbit monoclonal, #4858), phospho‐AKT (Ser 473) (D9E) XP(R) (rabbit mAb, #4060), phospho‐p44/42 MAPK (T202/Y204) (rabbit, #9101), total AKT (rabbit, #9272), total MAPK (mouse, #9107), total S6 (rabbit, #2217), Mcl‐1 (rabbit, #5453), Bcl‐xL (rabbit, #2764), Bcl‐2 (human specific, #2872) and beta‐tubulin (rabbit polyclonal, #2146). Anti‐GAPDH (14C10) (rabbit mAb, #2118) (Cell Signaling Technology) was used at a dilution of 1:3000.

### 2.6 AML patient cells

Methodology is described in detail in Appendix S1.

### 2.7 Drug combination studies

Methodology for drug combination studies^24^ is described in detail in Appendix S1.

### 2.8 Non‐invasive in vivo bioluminescence studies

All animal studies were performed according to protocols approved by the Dana‐Farber Cancer Institute's Institutional Animal Care and Use Committee.

Bioluminescence imaging was carried out as previously described.^25^ Briefly, for administration to female NSG mice (6‐8 weeks of age; The Jackson Laboratory, Bar Harbor, ME), virus‐ and *Mycoplasma*‐free SKNO‐1‐luc+ cells or OCI‐AML3‐luc+ cells were washed and resuspended in 1X PBS and administered via IV tail vein injection (2 × 10^6^ cells/250 µL PBS). A sample size of at least 8 mice per treatment group was chosen to ensure statistical significance. Anaesthetized mice were imaged 3 days after IV injection of cells to generate a baseline used to establish treatment cohorts and matched leukaemia burden (mice were randomized). Drug treatments were also started 3 days after inoculation. Total body luminescence was measured as previously described.^26^ Mice were treated with vehicle (n = 10) or midostaurin (100 mg/kg) (n = 10) for 28 days by oral gavage qD. 80 mg/kg was the tolerated dose for midostaurin purchased from MedChemExpress, LC Labs and BOC Sciences, whereas 100 mg/kg was the tolerated dose for midostaurin received from Novartis Pharma AG. For the SKNO‐1‐luc+ xenograft study, midostaurin stocks were obtained from Novartis Pharma AG, LC Labs, BOC Sciences and MedChemExpress. For the OCI‐AML3‐luc+ xenograft study, midostaurin stock was obtained from Novartis Pharma AG.

Midostaurin was formulated as a pre‐concentrate/microemulsion with 5% drug powder, 34% Vit E TPGS, 42.5% PEG400, 8.5% corn oil and 10% ethanol. The pre‐concentrate was then dissolved in purified water at a 24:76 ratio the day of treatment.

For all in vivo studies, *P* < .05 was considered to be statistically significant. The data had similar variance and met the assumptions of the tests carried out. For in vivo studies investigating the single‐agent effects of midostaurin, the Mann–Whitney test (two‐tailed) was carried out to assess differences in leukaemia burden between vehicle and drug‐treated mice and the Gehan‐Breslow‐Wilcoxon test was carried out for survival curve comparisons.

## 3 RESULTS

### 3.1 Comparison of anti‐proliferative effects of midostaurin and FLT3 inhibitors against wt FLT3‐expressing AML cell lines

Clinical trial results demonstrate efficacy of midostaurin in wt FLT3‐expressing AML.^6^, ^8^ Significantly, midostaurin and its major metabolites have been reported to have a number of kinase targets implicated in transformation and chemoresistance.^12^ Although considerable pre‐clinical and clinical data have been generated showing efficacy of midostaurin against mutant FLT3‐positive AML, comparatively little is known about the effectiveness of midostaurin against AML expressing wt FLT3 and other driver oncogenes. Midostaurin and other, more targeted FLT3 inhibitors were thus investigated to better understand important targets.

**Figure 1:**
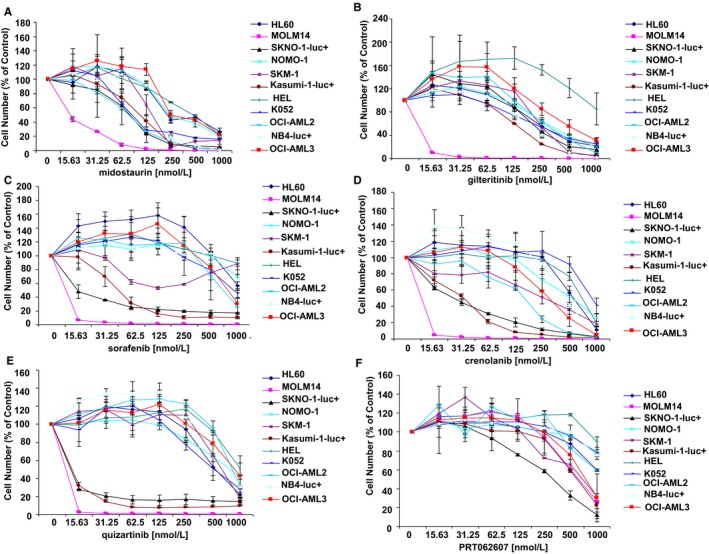
Comparison of effects of FLT3 inhibitors against the growth of human AML cell lines. Cells were treated for approximately three days. Targeted SYK inhibitor, PRT062607, was tested in parallel with the FLT3 inhibitors. (A‐F) Treatment of AML cell lines with midostaurin (A), gilteritinib (B), sorafenib (C), crenolanib (D), quizartinib (E) and PRT062607 (F)

We measured the efficacy of midostaurin and several advanced stage FLT3 inhibitors as single agents against human AML cell lines expressing either oncogenic FLT3 or wt FLT3. Midostaurin and the other FLT3 inhibitors, as expected, most potently suppressed the growth of FLT3‐ITD–positive MOLM14 cells; however, the inhibitors effectively killed wt FLT3‐expressing AML lines at submicromolar concentrations (Figure 1A‐E). As predicted, cell lines expressing an Asn822Lys mutation of KIT (SKNO‐1‐luc+ and Kasumi‐1‐luc+)^27^, ^28^ were particularly sensitive to FLT3 inhibitors that have been reported to display strong mutant KIT inhibitory activity, including sorafenib and quizartinib^29^, ^30^ (Figure 1C‐E). The targeted SYK inhibitor, PRT062607, was included as a negative control in that it displays no FLT3 inhibitory activity.^22^ As expected, mutant FLT3‐expressing MOLM14 cells were significantly growth‐inhibited by the panel of FLT3 inhibitors; however, PRT062607 showed much less potency against these cells (Figure 1F).

**Figure 2:**
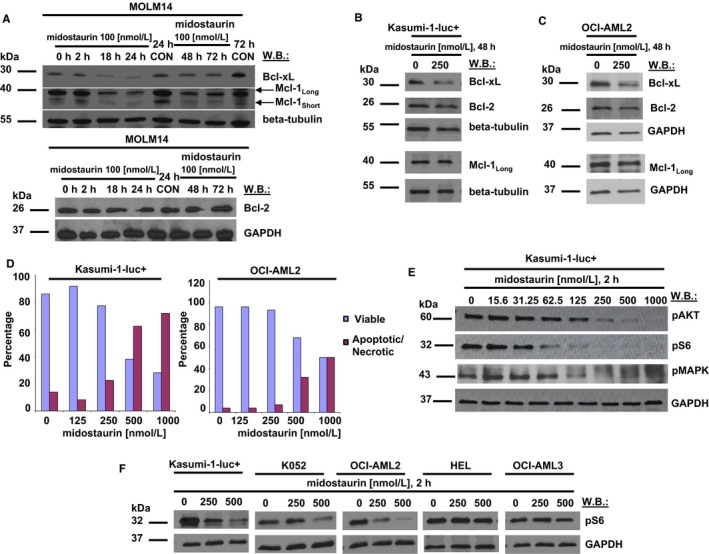
Effects of midostaurin on signalling molecules in wt FLT3‐expressing and mutant FLT3‐expressing cells. (A) Effects of midostaurin at 100 nmol/L on levels of Bcl‐xL, Bcl‐2 and Mcl‐1 in mutant FLT3‐expressing MOLM14 cells. (B, C) Effects of midostaurin on levels of Bcl‐xL, Bcl‐2 and Mcl‐1 in wt FLT3‐expressing Kasumi‐1‐luc+ cells (B) or OCI‐AML2 cells (C). (D) Induction of apoptosis by midostaurin in wt FLT3‐expressing AML cells. The apoptosis studies carried out for midostaurin‐treated Kasumi‐1‐luc+ cells and OCI‐AML2 cells were performed once each. The flow cytometry data from which the numerical values shown in the bar graphs were derived are now shown in Figure S1. (E) Effects of midostaurin at the indicated concentrations on phosphorylation of key molecules involved in PI3K/AKT and MAPK signalling in wt FLT3‐expressing Kasumi‐1‐luc+ cells. (F) Effects of midostaurin at the indicated concentrations on phosphorylation of S6 in wt FLT3‐expressing AML cell lines conferring differential sensitivity to midostaurin. WB = Western blot

To better understand the mechanism of midostaurin against AML cells expressing either mutated or wt FLT3, we next examined its effects on signalling molecules, including those implicated in inhibiting apoptosis as well as those contributing to cellular transformation. Consistent with the characterization of midostaurin as pro‐apoptotic,^5^ at a concentration of 100 nmol/L, midostaurin, in a time‐dependent manner, decreased levels of Mcl‐1 and Bcl‐xL in mutant FLT3‐expressing Ba/F3 and MOLM14 cells, with effects apparent within 24 hours (Figures 2A and S1A). For wt FLT3‐expressing Kasumi‐1‐luc+ cells and OCI‐AML2 cells, midostaurin modestly decreased levels of Bcl‐xL, however not Mcl‐1, at 250 nmol/L following 2 days of treatment (Figure 2B,C). Down‐regulation of Bcl‐xL by midostaurin correlated with induction of apoptosis in both cell lines (Figures 2D and [Link], [Link]B,C). Midostaurin inhibited phosphorylation of key mediators of PI3K/AKT and MAPK signalling, including AKT, S6 and MAPK, in a concentration‐dependent manner in wt FLT3‐expressing Kasumi‐1‐luc+ cells, with strong suppression of S6 and MAPK phosphorylation observed at 125 nmol/L and no effects on total AKT, S6 and MAPK protein levels (Figures 2E and S1D‐F). The inhibitory effects of midostaurin on phosphorylation of AKT, S6 and MAPK in wt FLT3‐expressing AML are similar to those observed with midostaurin‐treated, mutant FLT3‐positive MOLM14 cells; however, strong inhibition of these molecules was observed at concentrations less than 100 nmol/L in the mutant FLT3‐expressing cells (Figure S1G). Midostaurin also inhibited S6 phosphorylation in OCI‐AML2 cells in a concentration‐dependent manner, with robust inhibition observed at 500 nmol/L, although there was no inhibitory effect observed on AKT or MAPK phosphorylation in this cell line (Figure S1H). The utility of pS6 as a biomarker for the efficacy of midostaurin against wt FLT3‐expressing AML was investigated by comparing drug effects on pS6 in AML cell lines that were around 2‐fold or more sensitive to midostaurin (Kasumi‐1‐luc+, K052, OCI‐AML2) than other AML cell lines (HEL, OCI‐AML3). A decrease in pS6 was observed in midostaurin‐treated Kasumi‐1‐luc+, K052 and OCI‐AML2 at concentrations 250‐500 nmol/L (Figure 2F). In contrast, there was no decrease in pS6 in HEL or OCI‐AML3 cells treated at the same concentrations (Figure 2F). These results suggest that pS6 is a good biomarker for midostaurin activity and reflect the inhibition of MAPK signalling by midostaurin as a key component of its mechanism. Indeed, we have recently shown that pS6 is an excellent biomarker for activity of a targeted small molecule inhibitor of ERK1/2.^31^

**Figure 3:**
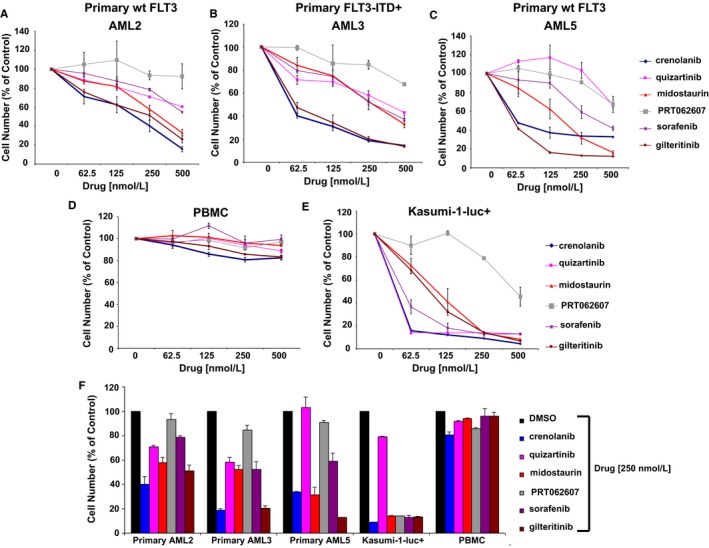
Anti‐proliferative effects of FLT3 inhibition and SYK inhibition on primary AML samples. (A‐C) Primary AML cells were treated for approximately three days. Targeted SYK inhibitor, PRT062607, was tested in parallel with the FLT3 inhibitors. Patient characteristics are summarized in Table S1. For AML2, no FLT3‐ITD mutation was detected. For AML3, a FLT3‐ITD mutation was detected (insertion of 12 nt 3′ to nt 1826, followed by a duplication of nt 1788‐1826 (total = 51 bp); the ITD is 7% of the total flt3 alleles in the specimen; this normally corresponds to 14% blasts. However, the specimen had 93% blasts on the day of this analysis. There is a minor clone with a 51bp ITD. For AML5, no FLT3‐ITD mutation was detected. (D) Treatment of normal PBMC cells with FLT3 inhibitors or PRT062607 for approximately 3 d. (E) Kasumi‐1‐luc+ cells were treated with FLT3 inhibitors or PRT062607 for approximately 3 d as a positive control. (F) Comparison of effects of inhibitors at 250 nmol/L against primary AML cells and normal PBMC cells, and against Kasumi‐1‐luc+ cells as a positive control

In addition to human cell lines, midostaurin and the other FLT3 inhibitors were also tested for efficacy against primary AML cells. The inhibitors were observed to kill primary human AML cells having a high (>90) per cent of blasts and expressing either wt FLT3 or oncogenic FLT3 in a concentration‐dependent fashion and to a greater extent than PBMCs from a normal donor, and with higher potency than PRT062607, used as a negative control because of its lack of FLT3 inhibitory activity (Figure 3A‐D,F). Results for FLT3 inhibitor‐treated Kasumi‐1‐luc+ cells are shown as a positive control (Figure 3E,F). These data, collectively, suggest that midostaurin and the other investigated FLT3 inhibitors are effective against wt FLT3‐expressing AML cells with concentration‐dependent killing at concentrations of 1 µmol/L or less.

**Figure 4:**
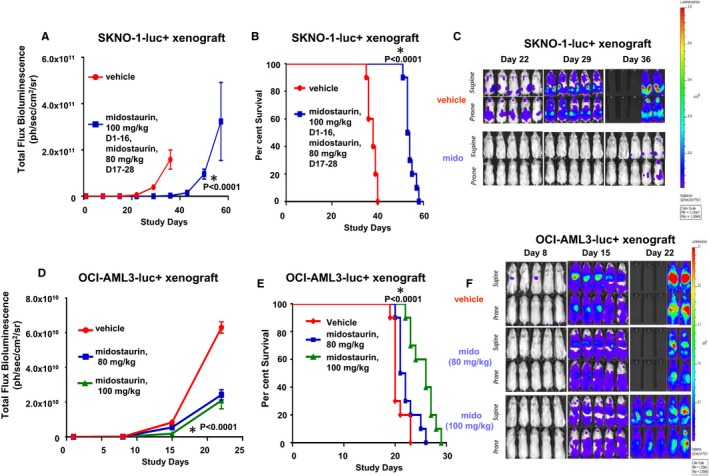
In vivo effects of midostaurin on growth of leukaemia cells in an SKNO‐1‐luc+ xenograft model and in an OCI‐AML3‐luc+ xenograft model. (A) Measure of leukaemia burden in vehicle‐ versus midostaurin‐treated mice for SKNO‐1‐luc+ xenograft model. For total flux bioluminescence, the Mann‐Whitney test (two‐tailed) was carried out. Vehicle versus midostaurin, *P* < .0001 (Day 8); *P* < .0001 (Day 15); *P* < .0001 (Day 22); *P* < .0001 (Day 29). (B) Measure of survival of vehicle‐ versus midostaurin‐treated mice for SKNO‐1‐luc+ xenograft model. The log‐rank (Mantel‐Cox) test and the Gehan‐Breslow‐Wilcoxon tests were carried out for survival curve comparisons. For both tests, *P* < .0001. (C) Effects of midostaurin in vivo against mice harbouring SKNO‐1‐luc+ cells: supine and prone (high scale). Days 22‐36. Representative images (n = 5). (D) Measure of leukaemia burden in vehicle‐ versus midostaurin‐treated mice for OCI‐AML3‐luc+ xenograft model. For total flux bioluminescence, the Mann‐Whitney test (two‐tailed) was carried out. Vehicle versus midostaurin (80 mg/kg, Day 15), *P* = 0656; vehicle versus midostaurin (100 mg/kg, Day 15), *P* < .0001; midostaurin (80 mg/kg, Day 15) versus midostaurin (100 mg/kg, Day 15), *P* = .0001. (E) Measure of survival of vehicle‐ versus midostaurin‐treated mice for OCI‐AML3‐luc+ xenograft model. The log‐rank (Mantel‐Cox) test and the Gehan‐Breslow‐Wilcoxon tests were carried out for survival curve comparisons. For both tests, *P* < .0001. For vehicle versus midostaurin (80 mg/kg), the log‐rank (Mantel‐Cox) test yielded a *P* value = .0547, and the Gehan‐Breslow‐Wilcoxon test yielded a *P* value = .0210. For midostaurin (80 mg/kg) versus midostaurin (100 mg/kg), the log‐rank (Mantel‐Cox) test yielded a *P* value = .0027, and the Gehan‐Breslow‐Wilcoxon test yielded a *P* value = .0025. For vehicle versus midostaurin (100 mg/kg), the log‐rank (Mantel‐Cox) test yielded a *P* value = .0001, and the Gehan‐Breslow‐Wilcoxon test yielded a *P* value = .0002. (F) Effects of midostaurin in vivo against mice harbouring OCI‐AML3‐luc+ cells. Supine and prone (high scale), Days 8, 15 and 22. Representative images (n = 4)

Having established in vitro the anti‐proliferative activity of midostaurin and other FLT3 inhibitors, we next asked whether midostaurin could disrupt the proliferative activity in in vivo models of wt FLT3‐expressing AML (Figure 4). We first tested engraftment of SKNO‐1‐luc+ and Kasumi‐1‐luc+ cells, both of which express the KIT‐N822K mutation as well as the t(8;21)(q22;q22) translocation that generates the *RUNX1‐RUNX1T1* fusion gene.^27^, ^28^ As engraftment of SKNO‐1‐luc+ cells in mice was strikingly more efficient than engraftment of Kasumi‐1‐luc+ cells (Figure S2A), it was decided to test the anti‐leukaemic activity of midostaurin against SKNO‐1‐luc+ cell growth in vivo*.* An OCI‐AML3‐luc+ xenograft model was also utilized to measure the in vivo efficacy of midostaurin; OCI‐AML3‐luc+ cells express *NRAS* Q61L and carry an NPM1 gene mutation (type A) and the DNMT3A R882C mutation.^32^, ^33^ Midostaurin significantly lowered leukaemia burden as a function of bioluminescence in mice (*P* < .0001 for SKNO‐1‐luc+ xenograft model and *P* < .0001 for OCI‐AML3‐luc+ xenograft model) (Figures 4A,D and S2B), and significantly increased median survival (*P* < .0001 for SKNO‐1‐luc+ xenograft model and *P* < .0001 for OCI‐AML3‐luc+ xenograft model) (Figure 4B,E). Representative mouse images for vehicle control and midostaurin treatment groups are shown in Figures 4C,F and S3. Midostaurin was well‐tolerated at 80 mg/kg in the SKNO‐1‐luc+ xenograft model and at 100 mg/kg in the OCI‐AML3‐luc+ xenograft model (Figure S2C,D). Taken together, these data suggest that wt FLT3‐expressing AML cells can be efficiently targeted in vivo by midostaurin.

### 3.2 SYK is a target of midostaurin but not of other FLT3 inhibitors in clinical development

The multi‐targeted nature and unique properties of midostaurin offer potential clinical benefits that other inhibitors may not, including suppression of kinases other than FLT3, such as mutant KIT, which play a role in aberrant signalling characteristic of the transformed phenotype or that are implicated in stromal cell–mediated chemoresistance.^12^ A recent study reported that SYK, a protein implicated in AML transformation and drug resistance, was among a panel of signalling molecules, including VEGFR2, LYN, IGF1R, RET, PDPK1 and TRKA, which were potently suppressed not only by midostaurin but also by its metabolites.^12^ Consistent with this, we have previously reported that SYK is a target of midostaurin.^22^

**Figure 5:**
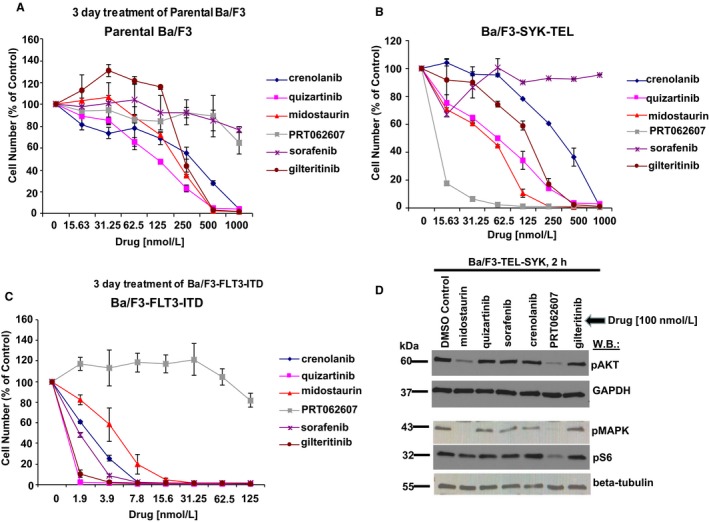
Comparison of effects of FLT3 inhibitors on growth of cells expressing activated SYK or oncogenic FLT3. (A‐C) Proliferation curves generated for parental Ba/F3 cells, Ba/F3‐SYK‐TEL cells and Ba/F3‐FLT3‐ITD cells treated for approximately three days with FLT3 inhibitors (crenolanib, quizartinib, midostaurin, sorafenib and gilteritinib) or the targeted SYK inhibitor, PRT062607. (D) Comparison of effects of FLT3 inhibitors on activation of signalling molecules downstream of activated SYK. PRT062607 was included as a positive control for SYK inhibition

We were interested in comparing the efficacy of midostaurin with other FLT3 inhibitors against cells driven by activated SYK to determine whether SYK inhibitory activity is unique to midostaurin or is a shared property of FLT3 inhibitors in general. We observed midostaurin to be intermediate in potency between the targeted SYK inhibitor, PRT062607, which suppressed proliferation to the highest extent, and other FLT3 inhibitors, including crenolanib, quizartinib, sorafenib and gilteritinib, which were less efficacious (Figures 5B and S4A). These results were consistent with SYK IC_50_s generated by the Thermo Fisher Scientific's SelectScreen™ Biochemical Kinase Profiling Service (10‐point titration assay): midostaurin (IC_50_ = 20.8 nmol/L), crenolanib (IC_50_ = 7970 nmol/L), gilteritinib (IC_50_ = 136 nmol/L) and sorafenib (IC_50_ > 10,000 nmol/L).

As expected, all of the FLT3 inhibitors strongly and selectively inhibited proliferation of Ba/F3‐FLT3‐ITD cells, with parental Ba/F3 cells showing less sensitivity (Figure 5A,C). Similarly anticipated, PRT062607 and the SYK inhibitor, entospletinib, which is currently under clinical investigation, were considerably less potent against Ba/F3‐FLT3‐ITD cells than Ba/F3‐SYK‐TEL cells and less effective against Ba/F3‐FLT3‐ITD cells than the FLT3 inhibitors (Figures 5C and S4B‐D). Consistent with the demonstrated potency against activated SYK‐expressing cells, midostaurin, similar to PRT062607, most potently inhibited phosphorylation of AKT and MAPK in Ba/F3 cells expressing and driven by activated SYK (Figure 5D). When effects of the FLT3 inhibitors against FLT3‐ITD–expressing cells were compared with effects against activated SYK‐expressing cells, there was a considerably smaller difference in the potency of midostaurin against the two oncoproteins than for the other FLT3 inhibitors (Figure S4E‐I). In contrast, anti‐proliferative effects of PRT062607 were much stronger against activated SYK‐expressing cells than FLT3‐ITD–expressing cells (Figure S4J).

Unlike midostaurin, the FLT3 inhibitors, gilteritinib, crenolanib, quizartinib and sorafenib, killed parental Ba/F3 cells and Ba/F3‐SYK‐TEL cells to similar extents (Figure S5). These results suggest that drug effects are unlikely to be because of targeted SYK inhibition and may be the result of general toxicity for these inhibitors. Similarly, Ba/F3‐SYK‐TEL cells treated with gilteritinib, crenolanib, quizartinib and sorafenib were more modestly rescued by IL‐3 than midostaurin‐treated cells (Figure S6). In contrast, IL‐3 impressively rescued the anti‐proliferative effects of FLT3 inhibitor treatment of Ba/F3‐FLT3‐ITD cells, suggestive of on‐target toxicity (Figure S7). Taken together, these data support the notion of targeted SYK suppression by midostaurin but not by the other FLT3 inhibitors.

### 3.3 Midostaurin potentiates effects of standard chemotherapy

**Figure 6:**
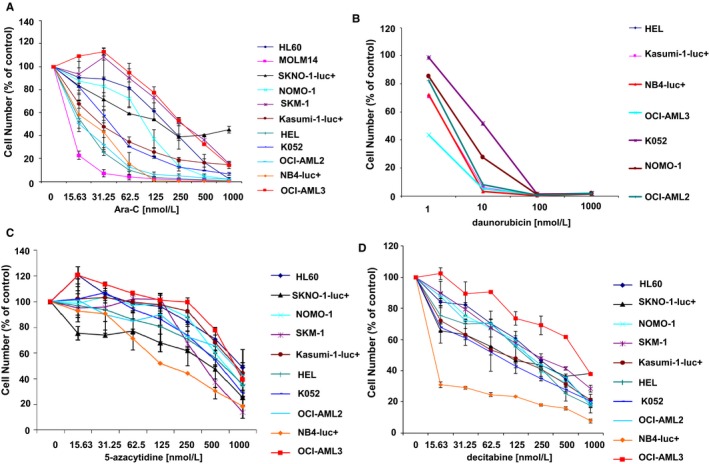
Effects of chemotherapeutic agents on proliferation of human AML cell lines. Treatment of AML lines with Ara‐C (A), daunorubicin (B), 5‐azacytidine (C) or decitabine (D). 3‐day assays. 5‐azacytidine and decitabine potencies shown here for OCI‐AML3 are similar to those previously published (Ref. [42])

Midostaurin has high synergizing potential and is able to positively combine with a number of agents, including 5‐azacytidine, decitabine and daunorubicin, as well as inhibitors of proviability signalling molecules such as the Bcl‐2 inhibitor ABT‐199 (venetoclax), the Bcl‐xL and Bcl‐2 inhibitor ABT‐263 (navitoclax), and the Mcl‐1 inhibitor S63845, against mutant FLT3‐expressing MOLM14 cells (Table 1; Figure S8). As such, we similarly investigated the effects of combining midostaurin with the same chemotherapy agents and proviability signalling molecule inhibitors against wt FLT3‐expressing AML cell lines that display a broad range of sensitivity to standard chemotherapy and targeted inhibitors such as venetoclax as single agents (Figures 6 and S9). With the exception of certain combination treatments, such as midostaurin combined with Ara‐C or daunorubicin against cell lines including OCI‐AML3 or NOMO‐1, K052 or NB4‐luc+, which were generally antagonistic, most combinations of midostaurin with chemotherapy agents or inhibitors of proviability signalling molecules showed positive (additive to synergistic) effects across a broad range of concentrations (Table 1; Figures [Link], [Link] and [Link], [Link], [Link], [Link], [Link]). Combinations of midostaurin and 5‐azacytidine or decitabine showed the highest degree of synergy across a wide range of concentrations and a number of wt FLT3‐expressing cell lines (Table 1; Figures [Link], [Link] and [Link], [Link], [Link], [Link], [Link]). Additive to synergistic effects was similarly observed for many of the combinations of midostaurin with inhibitors of proviability signalling molecules, with some exceptions showing antagonism, including the combination of midostaurin with venetoclax against OCI‐AML3 and HEL cells, and the combination of midostaurin with venetoclax or navitoclax against NOMO‐1 cells (Table 1; Figures [Link], [Link] and [Link], [Link], [Link], [Link], [Link]). Taken together, our data suggest that midostaurin is able to potentiate the effects of numerous other agents against wt FLT3‐expressing AML.

**Table 1:**
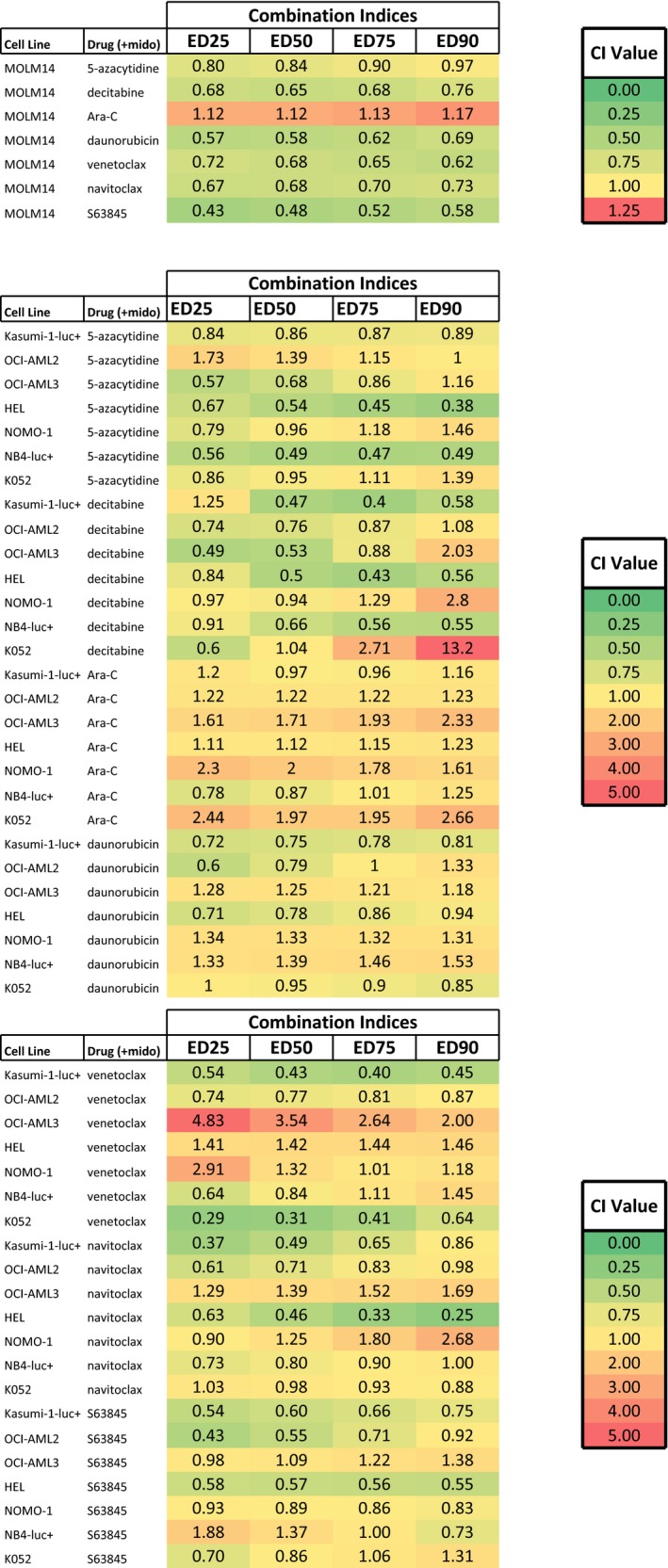
Combination indices generated by Calcusyn software for midostaurin combined with indicated chemotherapy agents or inhibitors of proviability signalling molecules against FLT3‐ITD–positive MOLM14 cells (panel 1, top); combination indices generated by Calcusyn software for midostaurin combined with indicated chemotherapy agents against wt FLT3 AML cell lines (panel 2, middle); combination indices generated by Calcusyn software for midostaurin combined with inhibitors of proviability signalling molecules against wt FLT3 AML cell lines (panel 3, bottom)

Note

Proliferation assays/combination studies were carried out for 3 d. Calcusyn combination indices can be interpreted as follows: CI values <0.10 indicate very strong synergism; values 0.10‐0.30 indicate strong synergism; values 0.30‐0.70 indicate synergism; values 0.70‐0.85 indicate moderate synergism; values 0.85‐0.90 indicate slight synergism; values 0.90‐1.10 indicate nearly additive effects; values 1.10‐1.20 indicate slight antagonism; values 1.20‐1.45 indicate moderate antagonism; values 1.45‐3.30 indicate antagonism; values 3.30‐10.0 indicate strong antagonism; values >10.0 indicate very strong antagonism.

## 4 DISCUSSION

Midostaurin has a potentially useful combination of targets that are involved in multiple key signalling pathways, and this multi‐targeted behaviour may account for some of the observed efficacy of the inhibitor against numerous AML genotypes. In addition, considering that overexpression of wt FLT3 is characteristic of the majority of AML cases, the importance of inhibiting wt FLT3 may have been previously unappreciated.

Early investigations revealed the potential of AML patients without mutations in FLT3 to be clinically responsive to midostaurin and are consistent with the results presented herein using our cell line–based and murine models of wt FLT3 AML.^8^, ^9^ Specifically, in a Phase IIb study, haematologic improvement was observed in 16/35 AML patients harbouring FLT3 mutations and 20/57 AML patients expressing wt FLT3, and more than half of patients exhibited peripheral blood or bone marrow blast reduction independent of FLT3 mutational status, although with a higher overall incidence observed for mutant FLT3‐positive patients.^8^ Similarly, in a Phase I study, 100 mg midostaurin treatment twice daily led to complete responses in 8/23 wt FLT3‐expressing patients and 5/56 mutant FLT3‐expressing patients, with 9 wt FLT3 patients and 2 mutant FLT3 patients surviving over 4 years.^9^ For patients treated with 50 mg midostaurin twice a day, combined with or following chemotherapy, the complete response rate was 74% for wt FLT3 AML and 92% for mutant FLT3 AML.^9^

Clinical benefit was also observed in the large Phase III RATIFY trial in AML patients regardless of their FLT3 mutant: FLT3 wt ratio and the type of FLT3 mutation they harboured.^6^ Based on this, a multinational, randomized Phase III clinical trial has commenced, investigating the efficacy of midostaurin in combination with chemotherapy and also as single‐agent maintenance therapy in newly diagnosed AML patients harbouring wt FLT3.^34^

Treatment of AML patients with a FLT3 inhibitor as a single agent is not likely to lead to a complete remission and therefore combination with other agents can potentially provide more clinical benefit, as has previously been observed.^6^ A benefit of combined use of FLT3 inhibitors with other agents is overriding of some forms of drug resistance. For example, midostaurin, through inhibition of the drug transport function of the ATP‐binding cassette (ABC) protein ABCB1, resensitizes multidrug‐resistant cancer cells to standard chemotherapeutic agents.^35^

Whereas overall response rates of first‐generation FLT3 inhibitors such as midostaurin as single agents have ranged from 0% to 3%,^8^ overall response rates have been 20%‐40% when these inhibitors were combined with standard chemotherapy.^36^, ^37^ This is compared to an overall response rate of second‐generation FLT3 inhibitors as single agents of 40%‐50%; however, duration of response is short and haematologic recovery is incomplete for these inhibitors.^38^ Other limitations of second‐generation FLT3 inhibitors include a short (6‐8 hours) half‐life for crenolanib, requiring thrice‐daily dosing, and a lack of activity of quizartinib against FLT3‐TKD mutants such as those occurring at the D835 or F691 residues.^17^

We demonstrated the ability of midostaurin to behave as a chemosensitizer of numerous chemotherapeutic and targeted agents, such inhibitors of proviability signalling molecules, against AML cells regardless of their FLT3 mutational status. As midostaurin causes loss of proviability signalling molecules, such as Mcl‐1 and Bcl‐xL, we propose that the direct effect of midostaurin on the apoptotic machinery of AML cells (as shown in Figure 2), in addition to the drug's broad spectrum of targets that includes molecules such as KIT, PDGFR, VEGFR and SYK, may contribute to the widespread synergizing potential of midostaurin. Synergy was observed between midostaurin coupled with venetoclax, navitoclax or S63845; the panel of wt FLT3‐expressing human AML cell lines we tested included those showing either high sensitivity or relatively high resistance to venetoclax as a single agent as has been previously reported.^39^ We generally observed some combinations between midostaurin and standard chemotherapeutic agents to show stronger synergy than others, with comparatively weaker combination effects observed for the simultaneous administration of midostaurin+ Ara‐C against both mutant FLT3‐ and wt FLT3‐expressing cells. Sequential versus simultaneous administration of midostaurin and Ara‐C against Ba/F3‐FLT3‐ITD‐luc+ cells led to additive to synergistic effects that were generally comparable (Figure [Link], [Link]). Our findings are consistent with a report of variable results for Ara‐C+ midostaurin‐treated AML cell lines and patient samples, with antagonistic effects observed frequently in wt FLT3‐expressing cells (90%) and less so in FLT3‐ITD–positive cells (60%), with little effect of the timing or sequence of the drug administration.^40^

We examined FLT3 levels in all of the human AML lines used for our study (Figure [Link], [Link], [Link], [Link]), and we observed variable expression of FLT3, ranging from no detectable FLT3 protein expression in HL60 cells to high expression in cell lines such as SKNO‐1‐luc+ and OCI‐AML2 (highest FLT3 expression was observed in FLT3‐ITD–positive MOLM14 cells). FLT3‐null cells, such as HL60, showed moderate, submicromolar, sensitivity to midostaurin (IC50 near 250 nmol/L; Figure 1A), which was similar to the sensitivity of other cell lines with detectable FLT3, such as NOMO‐1, OCI‐AML3 and HEL. If FLT3 null patients, similar to the FLT3 null HL60 cell line model, respond to midostaurin, then this could be attributed to the broad‐spectrum activity of midostaurin, independent of FLT3 as a target.

We also tested the responsiveness of the AML cell lines to midostaurin when cultured in the presence versus the absence of FLT3 ligand, to investigate whether cell line sensitivity to midostaurin correlated with the level of FLT3 expression in the different cell lines. As expected, the presence of FLT3 ligand did not appear to significantly affect cell proliferation for null FLT3‐ or wt FLT3‐expressing AML lines treated with midostaurin, and the high or low expression of FLT3 in the different cell lines did not seem to affect their sensitivity to midostaurin in the presence of FLT3 ligand (Figure [Link], [Link], [Link], [Link]).

Encouraging results were recently reported for midostaurin in a ‘real‐world’ setting, namely higher remission rates in midostaurin‐treated patients that translated into an increased rate of transplantation in first remission.^41^ These promising clinical responses, coupled with pre‐clinical and clinical studies suggesting efficacy of midostaurin in mutant FLT3 as well as non‐mutated FLT3‐expressing patients, strongly support the investigation of midostaurin as a therapeutic for a more general population of AML patients.

## CONFLICT OF INTEREST

James D. Griffin receives funding and has received a royalty payment from Novartis Pharmaceuticals and receives funding from Eli Lilly and Company. Nathanael Gray is a founder, science advisory board member (SAB) and equity holder in Gatekeeper, Syros, Petra, C4, B2S and Soltego. The Gray Lab receives or has received research funding from Novartis, Takeda, Astellas, Taiho, Janssen, Kinogen, Voronoi, Her2llc, Deerfield and Sanofi. Ellen Weisberg has received a royalty payment from Novartis Pharmaceuticals. Richard Stone does ad hoc consulting for and receives clinical research support to Dana‐Farber Cancer Institute from the following companies: AbbVie, Agios, Arog and Novartis. He does ad hoc consulting for the following companies: Astrazeneca, Cornerstone, Jazz, Daiichi Sankyo, Otsuka/Astex, Pfizer and Stemline. He is on the Advisory Board of the following companies: Actinium, Amgen, Astellas and Macrogenics. He is on the Data Safety and Monitoring Board for the following companies: Argenx, Celgene and Takeda. He is an ad hoc consultant and on the Steering Committee and Data Safety and Monitoring Board for Celgene.

## AUTHOR CONTRIBUTIONS

All authors listed have (a) made substantial contributions to research design, or the acquisition, analysis or interpretation of data and (b) drafted the paper or revised it critically. All authors have approved the submitted and final version of this manuscript.

## Data Availability

The data that support the findings of this study are available from the corresponding author upon reasonable request.
