# Supplementary material for: Effects of the multi‐kinase inhibitor midostaurin in combination with chemotherapy in models of acute myeloid leukaemia
Source: J Cell Mol Med. 2020 Jan 22;24(5):2968–80. doi: 10.1111/jcmm.14927 (PMC7077552; doi:10.1111/jcmm.14927)
Supplement: Supplementary file 1 [file JCMM-24-2968-s001.doc]

Supplementary Materials and Methods

*Cell Lines*

Cell lines used in this study were cultured with 5% CO_2_ at 37°C, at a concentration of 2×10^5^ to 5×10^5^ in RPMI (Thermo Fisher Scientific (Waltham, MA)) with 10% fetal bovine serum (FBS) and 1% penicillin/streptomycin. Media for SKNO-1-luc+ was supplemented with 2 ng/mL GM-CSF. Parental Ba/F3 cells were cultured in RPMI with 10% FBS and supplemented with 2% L-glutamine and 1% penicillin/streptomcyin, and 20% WEHI (as a source of IL-3).

Human cell lines were authenticated within 6 months of manuscript preparation through cell line short tandem repeat (STR) profiling (DDC Medical, Fairfield, OH and Molecular Diagnostics Laboratory, Dana-Farber Cancer Institute, Boston, MA). All cell lines tested matched >80% with lines listed in the ATCC or DSMZ Cell Line Bank STR and were confirmed to be virus- and *Mycoplasma*-free.

*Cell proliferation studies and apoptosis studies*

The Trypan blue exclusion assay previously described (ref. 5), was utilized for cell seeding. CellTiter-Glo (Promega, Madison, WI) was used for proliferation studies according to manufacturer instructions. Cell viability is shown in graphs as the percentage of control (untreated) cells; error bars represent the standard deviation for each data point. Apoptosis was determined using the Annexin-V-Fluos Staining Kit (Boehringer Mannheim, Indianapolis, IN), as per manufacturer instructions.

*Drug combination studies*

For drug combination assays, we initially quantified cells for seeding using the Trypan Blue exclusion assay. The CellTiter-Glo protocol (per manufacturer's instructions) was then carried out for proliferation studies. Single agents were added simultaneously at fixed ratios to cells. Cell viability was subsequently expressed as a function of growth affected, drug-treated versus DMSO control cells and data were analyzed by Calcusyn software (Biosoft, Ferguson, MO and Cambridge, UK). This software, which is based on isobologram generation (ref. 24), was used for measurement of synergy or antagonism. This approach uses the median effect principle to quantify drug combination effects to assess whether or not they are greater than those expected from a simple addition of the single agent effects. Following estimation of the ED_50_ or IC_50_ of each drug, combinations were analyzed where the concentrations are fractions or multiples of the ED/IC_50_. Combination indices or values generated by the Calcusyn software are either less than one (indicative of synergy) or greater than one (indicative of antagonism).

*Chemical compounds and biologic reagents*

For *in vitro* studies, midostaurin, crenolanib, quizartinib, 5-azacytidine and decitabine were purchased from Haoyuan chemexpress (Shanghai, China). Ara-c and daunorubicin hydrochloride were purchased from Sigma-Aldrich (Milwaukee, WI). Sorafenib was purchased from LC Laboratories (Woburn, MA). Gilteritinib (Hydrochloride) was purchased from Chemietek (Indianapolis, IN). Entospletinib, PRT062607, ABT-199 (venetoclax; Venclexta), and ABT-263 were purchased from Selleckchem (Houston, TX). S63845 was purchased from Active Biochem LTD (Kowloon, Hong Kong). All drugs were dissolved in DMSO to obtain a 10 mM stock solution. Serial dilutions were then made, to obtain final dilutions for cellular assays with a final concentration of DMSO not exceeding 0.1%.

*AML patient cells*

Mononuclear cells were isolated from peripheral blood or bone marrow samples from AML patients identified as harboring mutant or wt FLT3 by density gradient centrifugation through Ficoll-Paque Plus (Amersham Pharmacia Biotech AB, Uppsala, Sweden) at 2000 rpm for 30 minutes, followed by two washes in 1X PBS. All samples were obtained under approval of the Dana Farber Cancer Institute Institutional Review Board. Informed consent was obtained from all subjects.

*Testing the growth of Kasumi-1-luc+ and SKNO-1-luc+ as disseminating models in NSG mice*

All animal studies were performed according to protocols approved by the Dana-Farber Cancer Institute's Institutional Animal Care and Use Committee.

Bioluminescence imaging was carried out as previously described (ref 25). Briefly, for administration to female NSG mice (6-8 weeks of age; The Jackson Laboratory, Bar Harbor, Maine), virus- and *Mycoplasma*-free SKNO-1-luc+ or Kasumi-1-luc+ cells were washed and resuspended in 1X PBS and administered via IV tail vein injection (2 X 10^6^ cells/250 μl PBS). Groups were divided into sample sizes of 5 mice per group. Mice were monitored for a total of 19 days and leukemia burden was measured by non-invasive bioluminescence imaging.
