# Supplementary figures and images for: Effects of the multi‐kinase inhibitor midostaurin in combination with chemotherapy in models of acute myeloid leukaemia
Source: J Cell Mol Med. 2020 Jan 22;24(5):2968–80. doi: 10.1111/jcmm.14927 (PMC7077552; doi:10.1111/jcmm.14927)

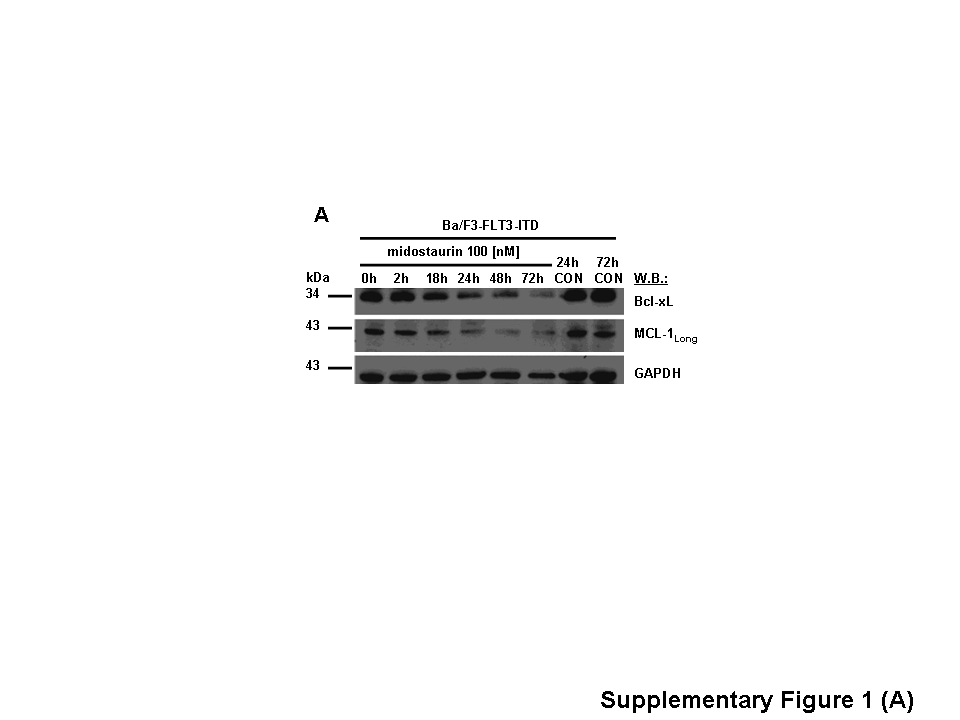

Supplement: Supplementary file 2 [file JCMM-24-2968-s002.TIF]

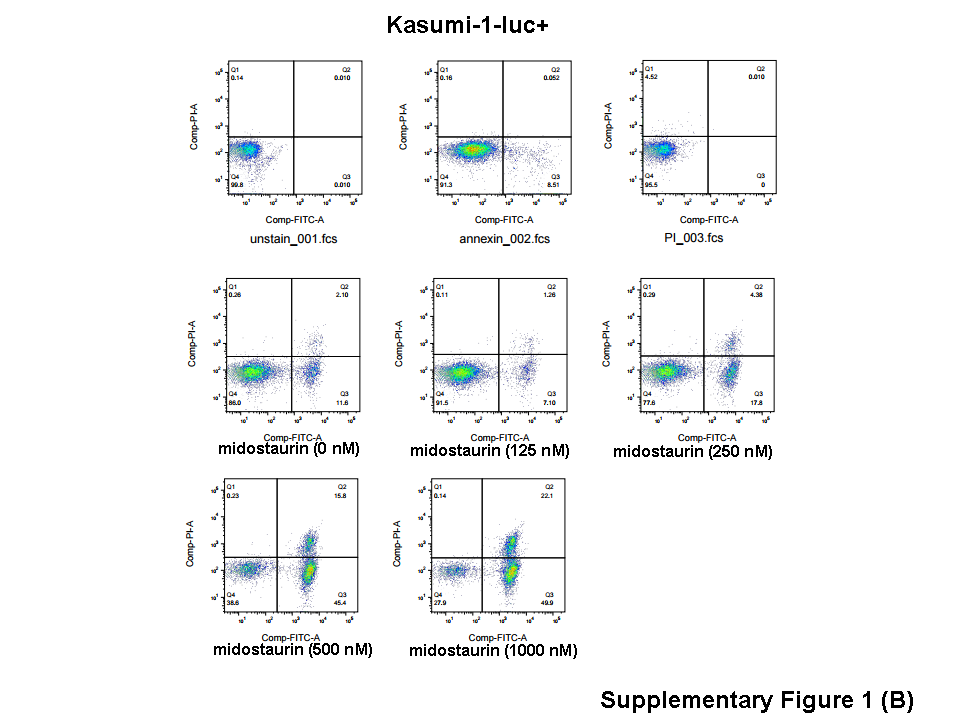

Supplement: Supplementary file 3 [file JCMM-24-2968-s003.TIF]

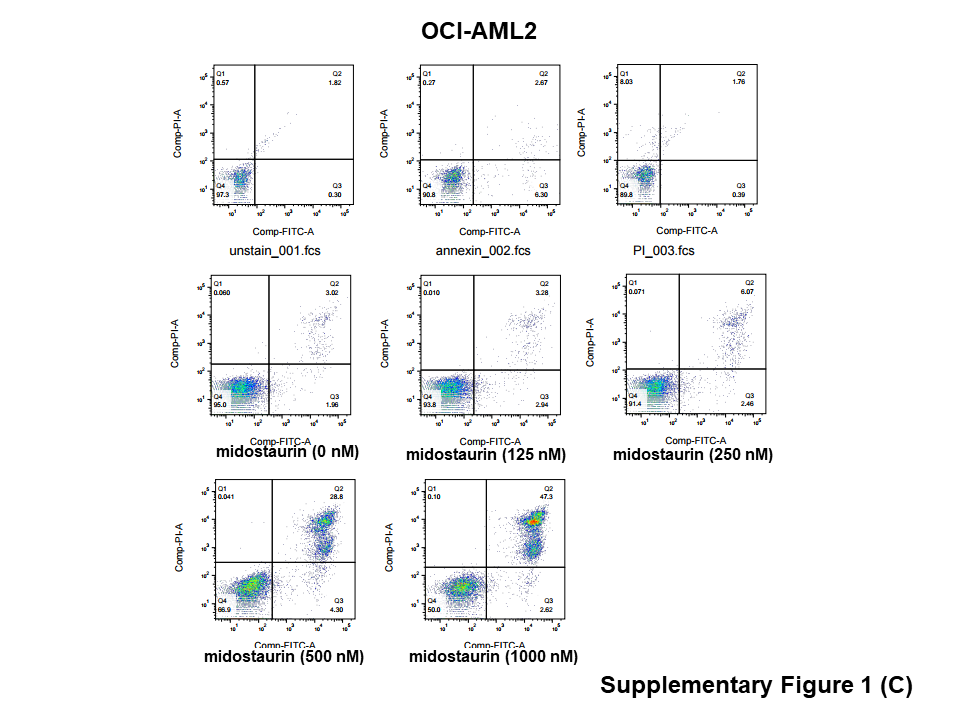

Supplement: Supplementary file 4 [file JCMM-24-2968-s004.TIF]

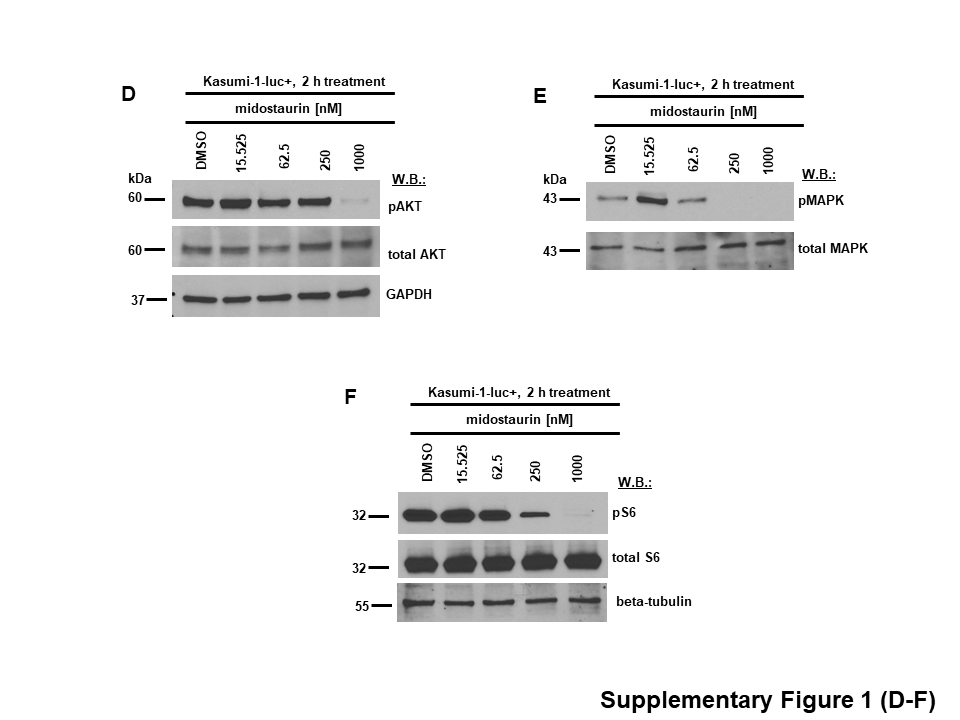

Supplement: Supplementary file 5 [file JCMM-24-2968-s005.TIF]

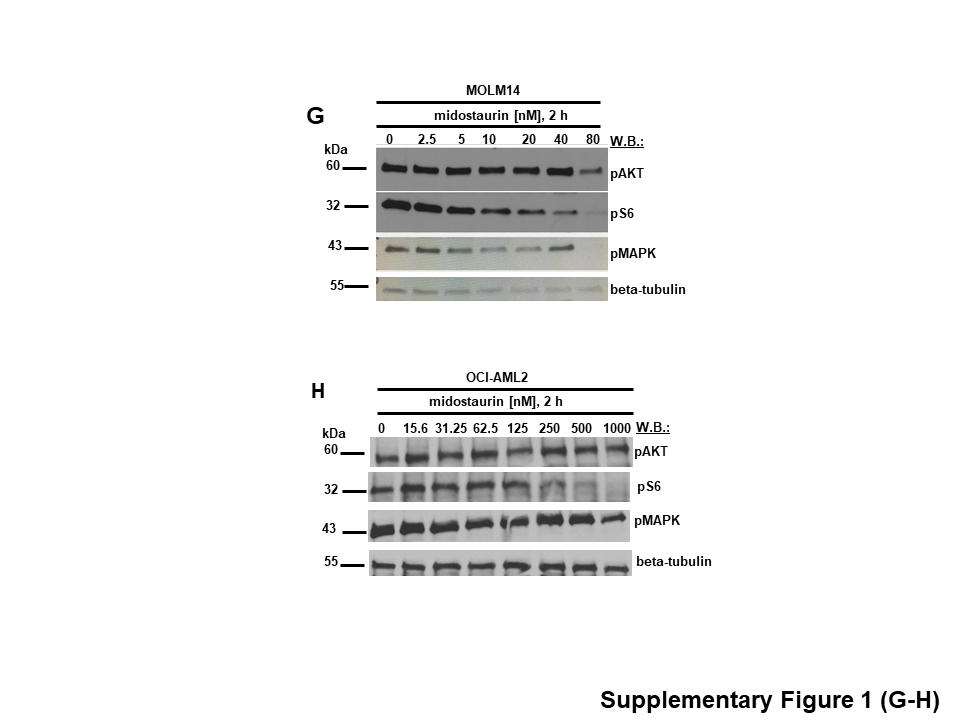

Supplement: Supplementary file 6 [file JCMM-24-2968-s006.TIF]

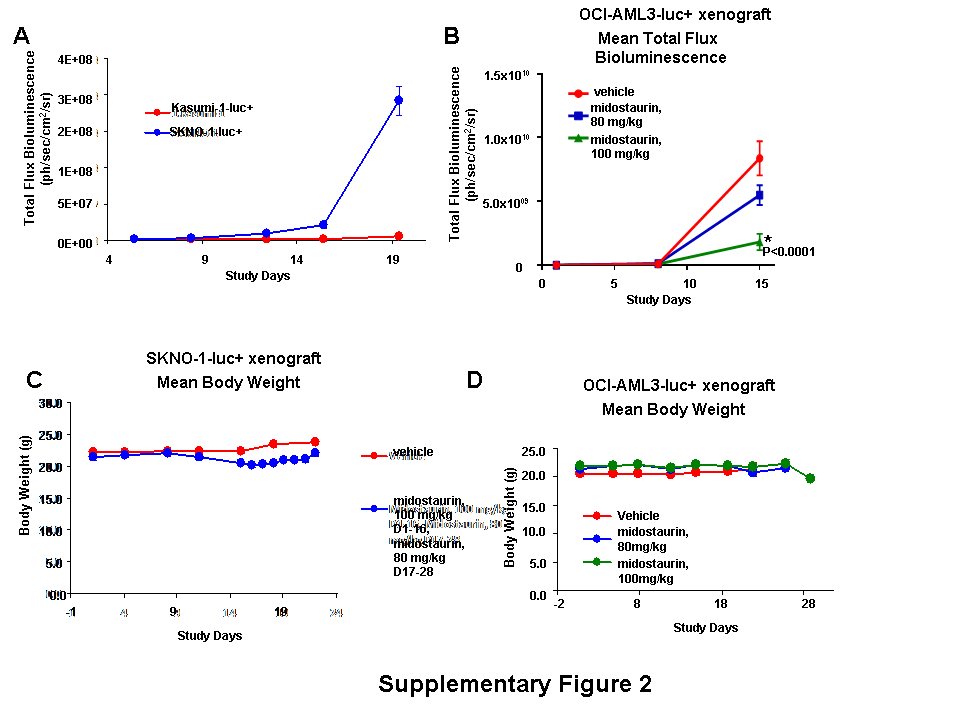

Supplement: Supplementary file 7 [file JCMM-24-2968-s007.TIF]

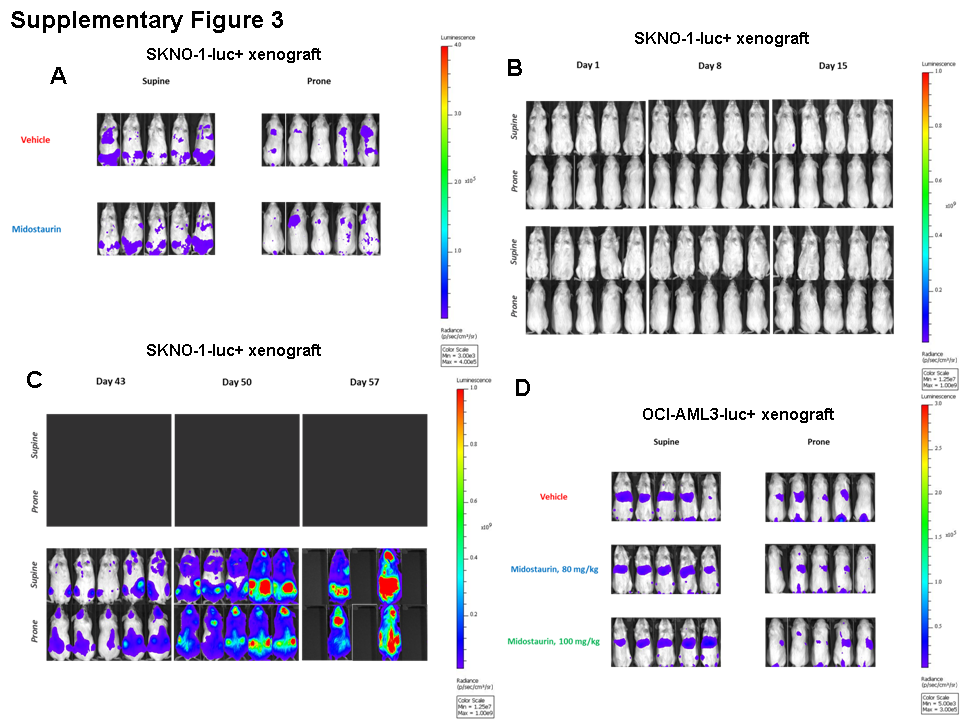

Supplement: Supplementary file 8 [file JCMM-24-2968-s008.TIF]

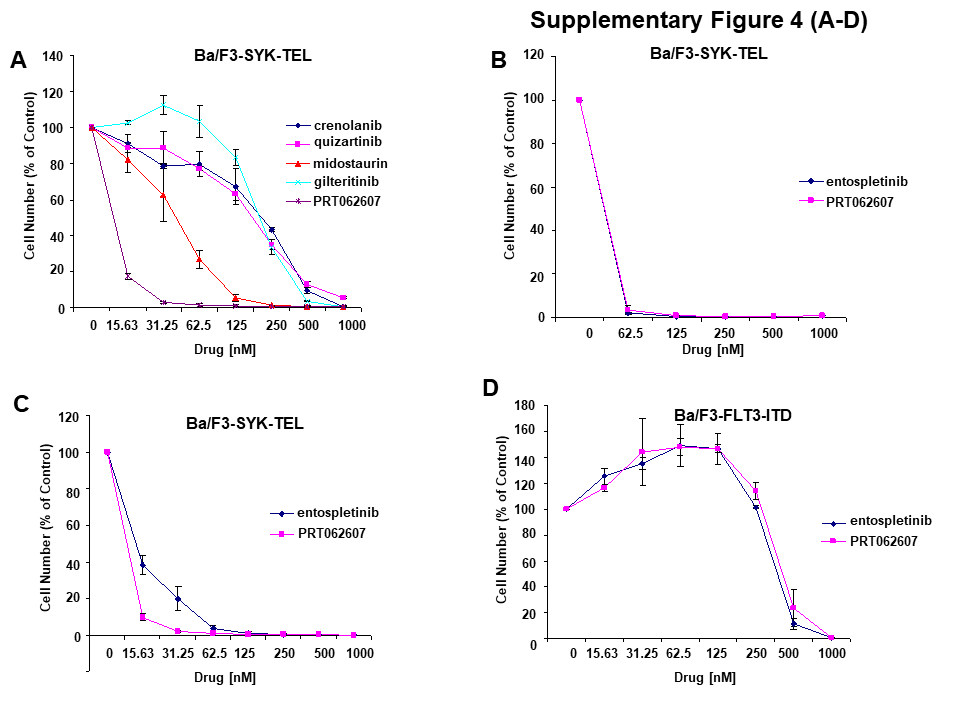

Supplement: Supplementary file 9 [file JCMM-24-2968-s009.TIF]

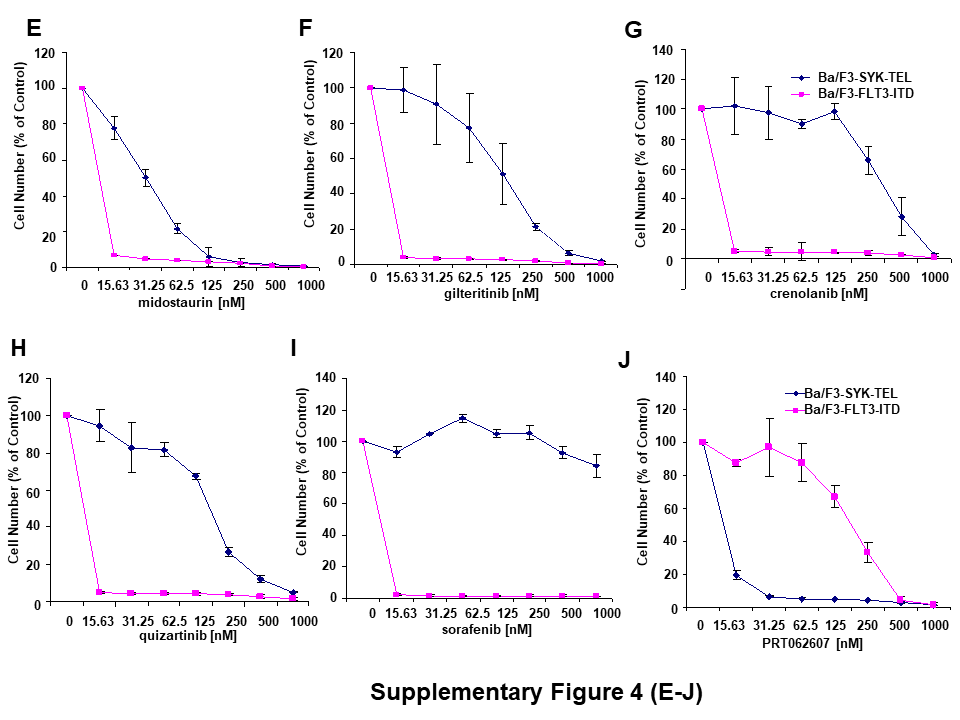

Supplement: Supplementary file 10 [file JCMM-24-2968-s010.TIF]

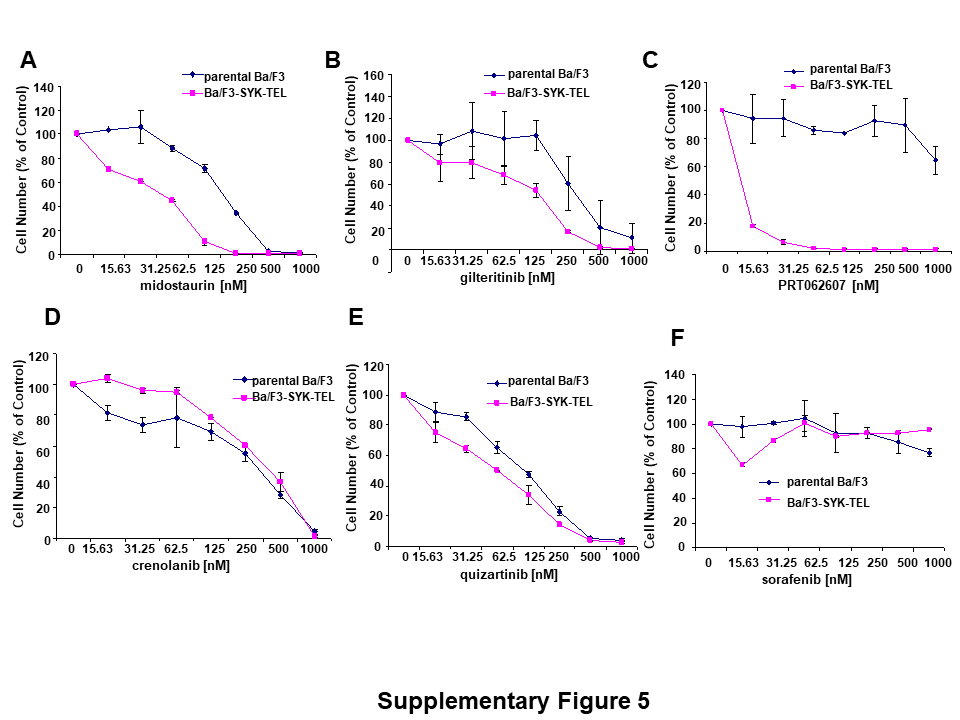

Supplement: Supplementary file 11 [file JCMM-24-2968-s011.TIF]

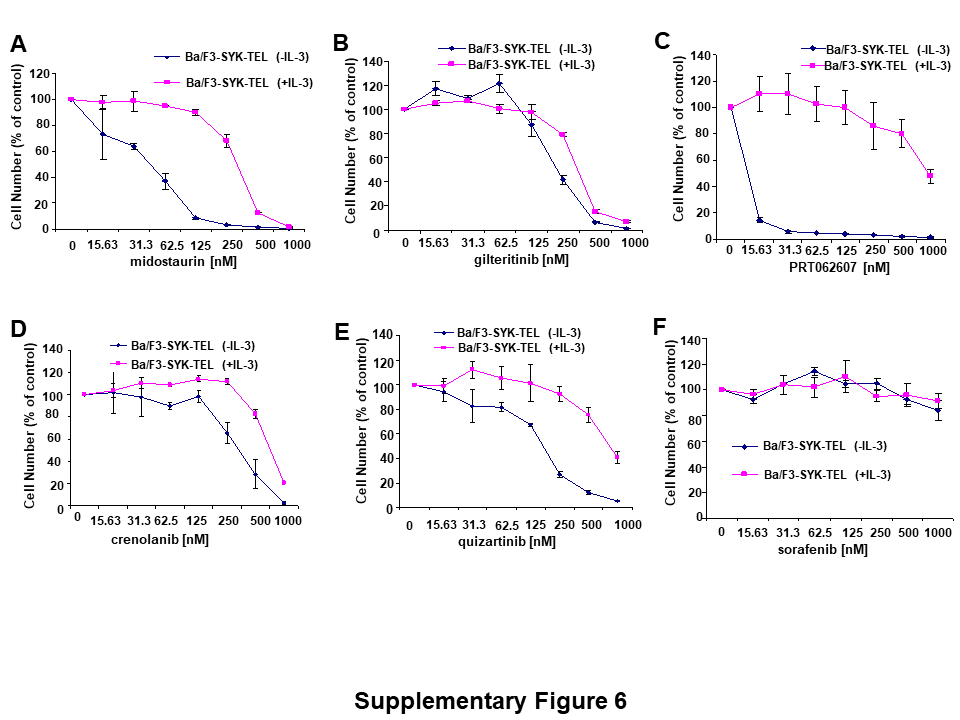

Supplement: Supplementary file 12 [file JCMM-24-2968-s012.TIF]

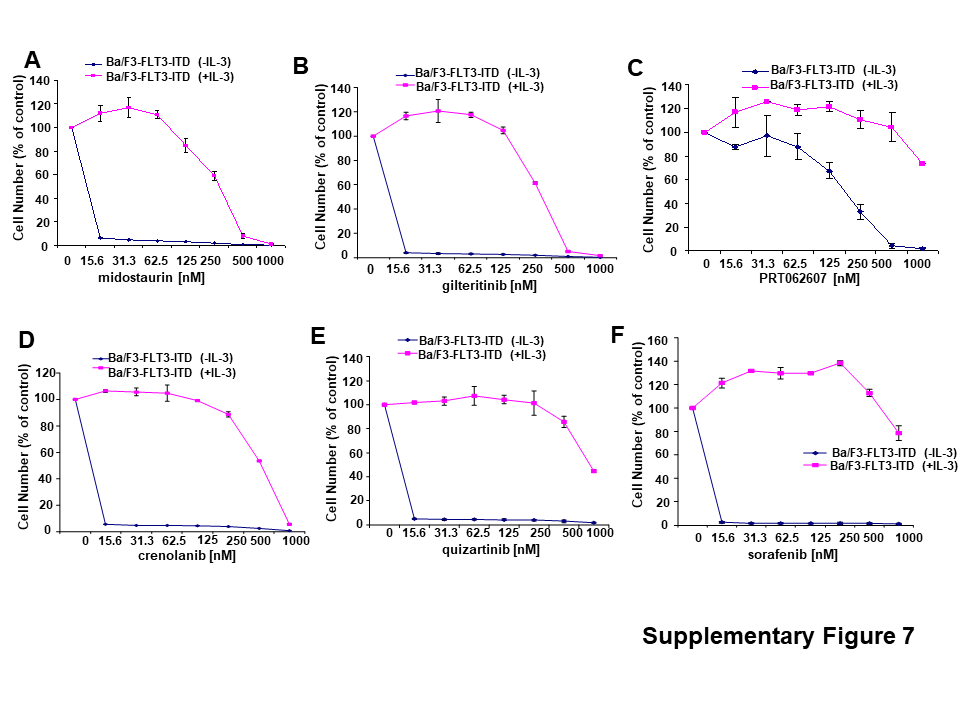

Supplement: Supplementary file 13 [file JCMM-24-2968-s013.TIF]

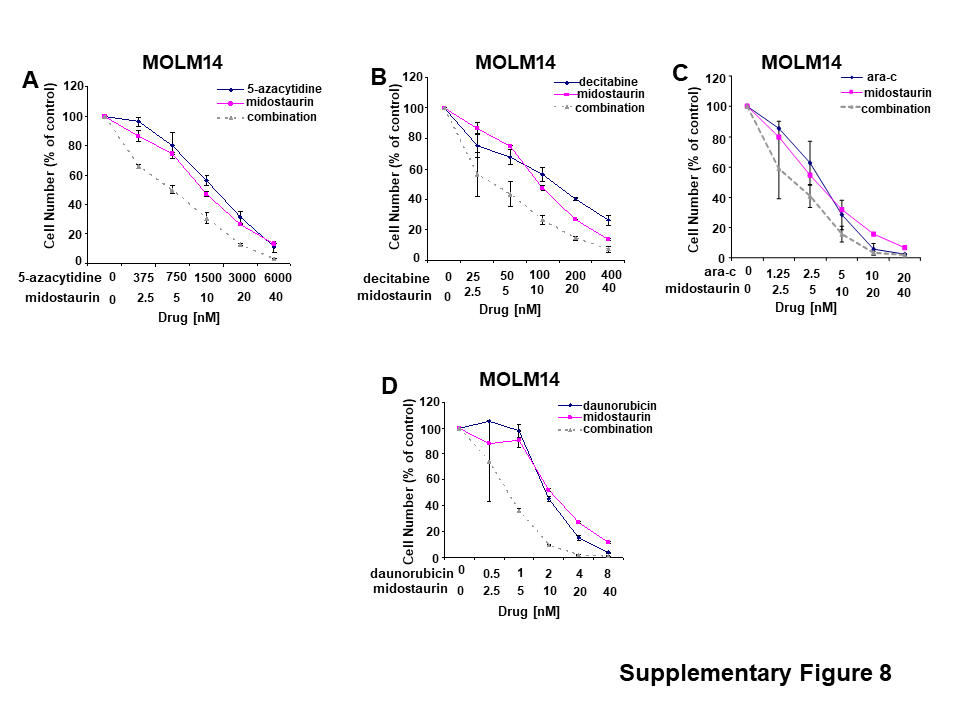

Supplement: Supplementary file 14 [file JCMM-24-2968-s014.TIF]

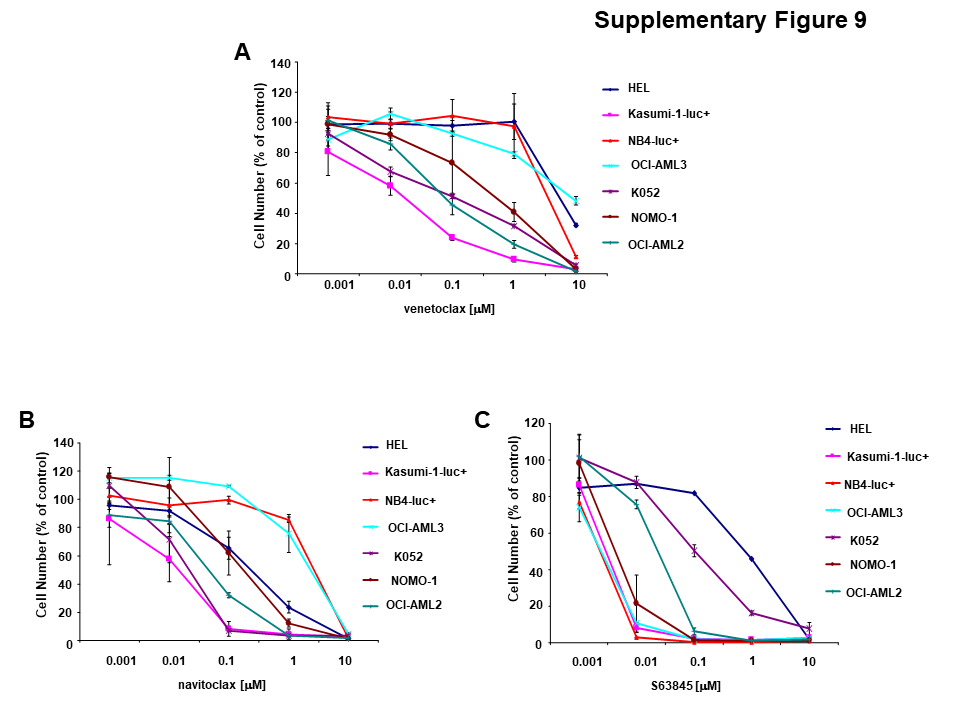

Supplement: Supplementary file 15 [file JCMM-24-2968-s015.TIF]

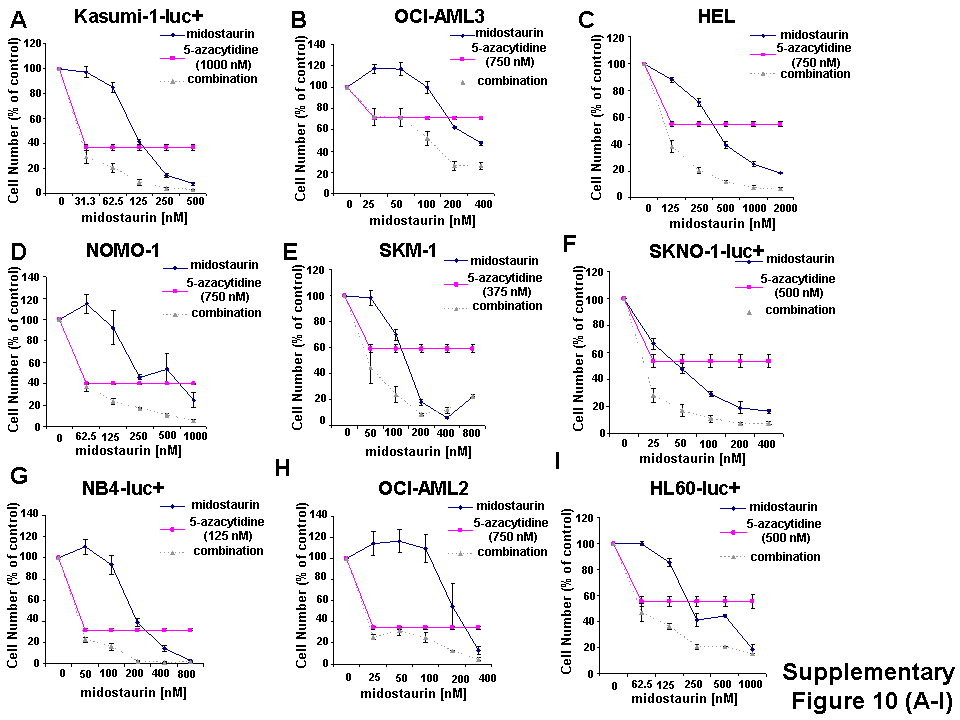

Supplement: Supplementary file 16 [file JCMM-24-2968-s016.TIF]

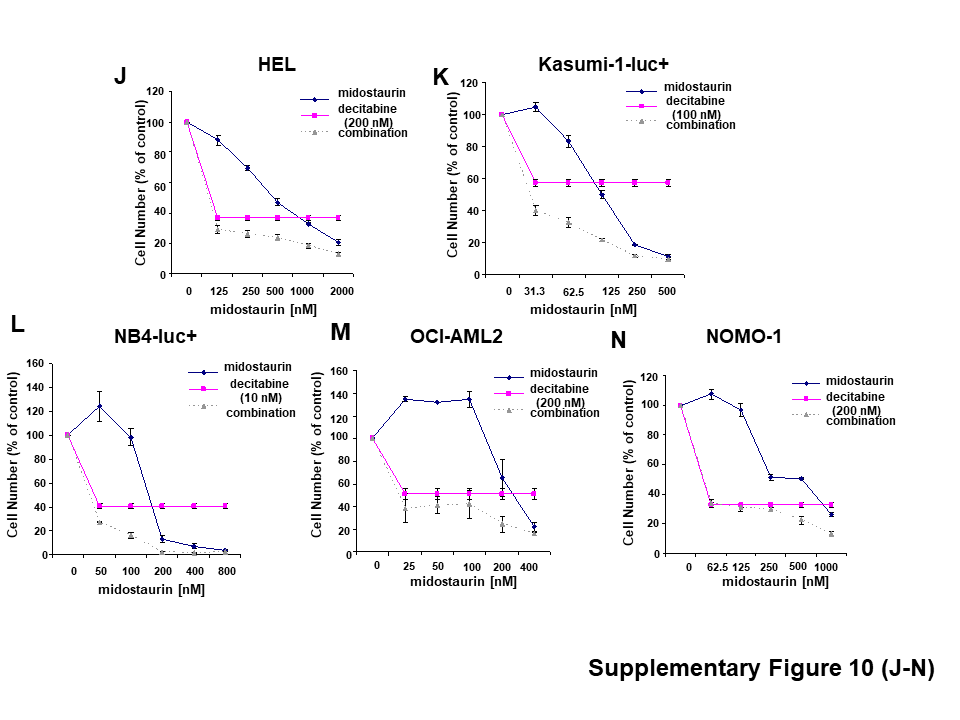

Supplement: Supplementary file 17 [file JCMM-24-2968-s017.TIF]

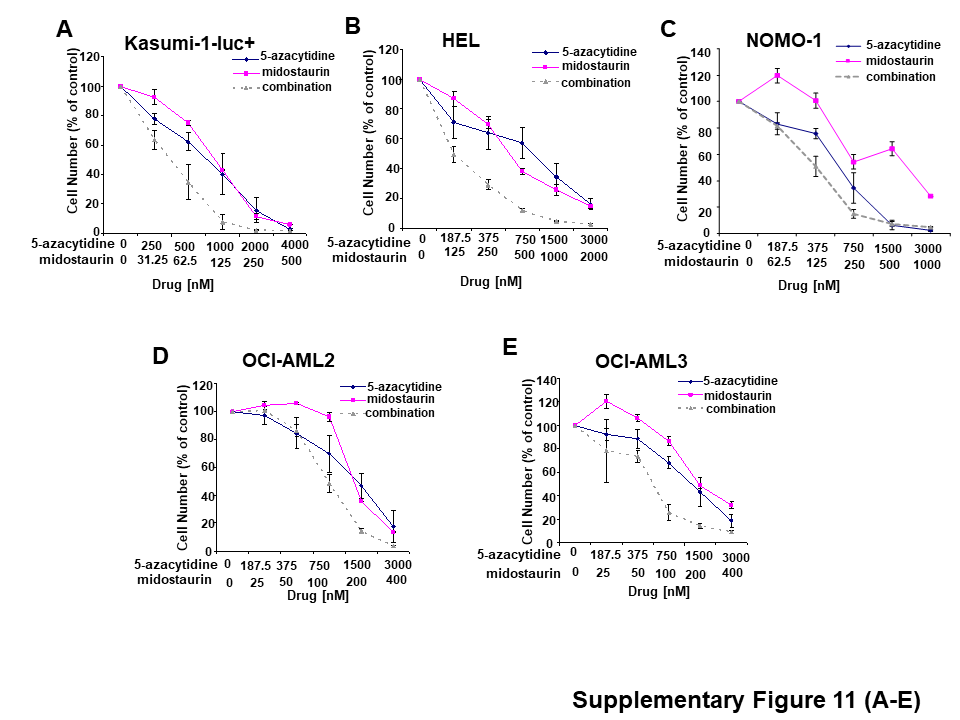

Supplement: Supplementary file 18 [file JCMM-24-2968-s018.TIF]

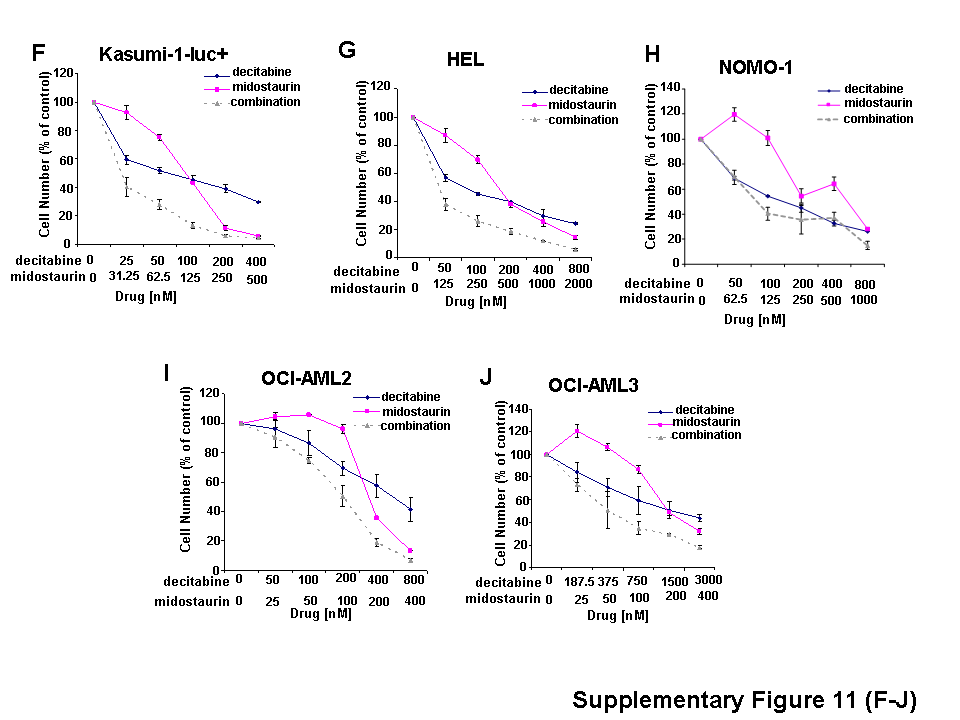

Supplement: Supplementary file 19 [file JCMM-24-2968-s019.TIF]

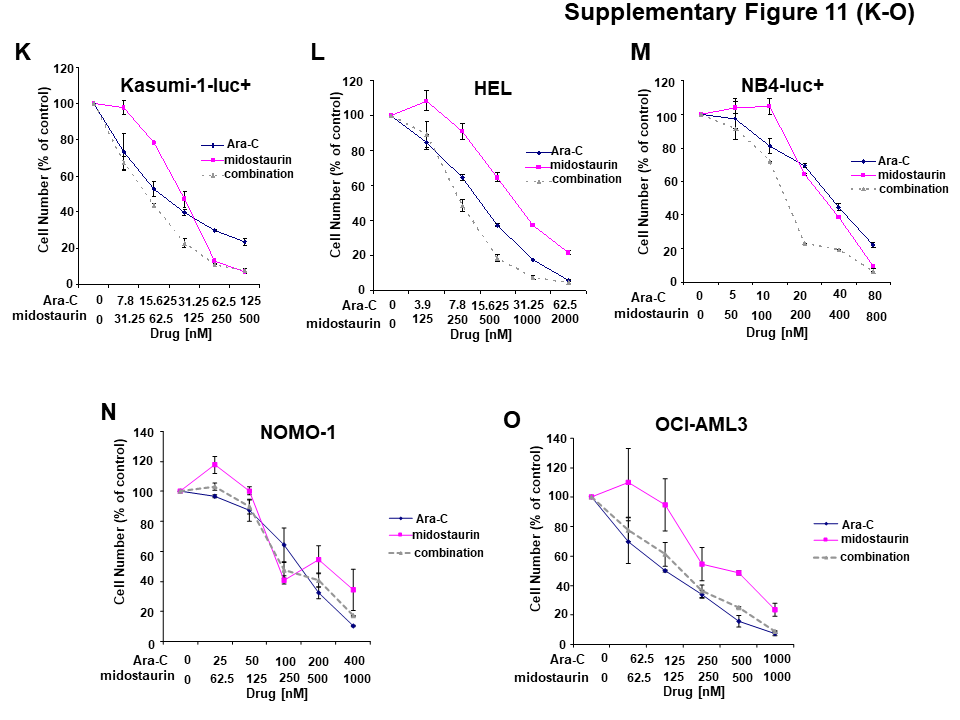

Supplement: Supplementary file 20 [file JCMM-24-2968-s020.TIF]

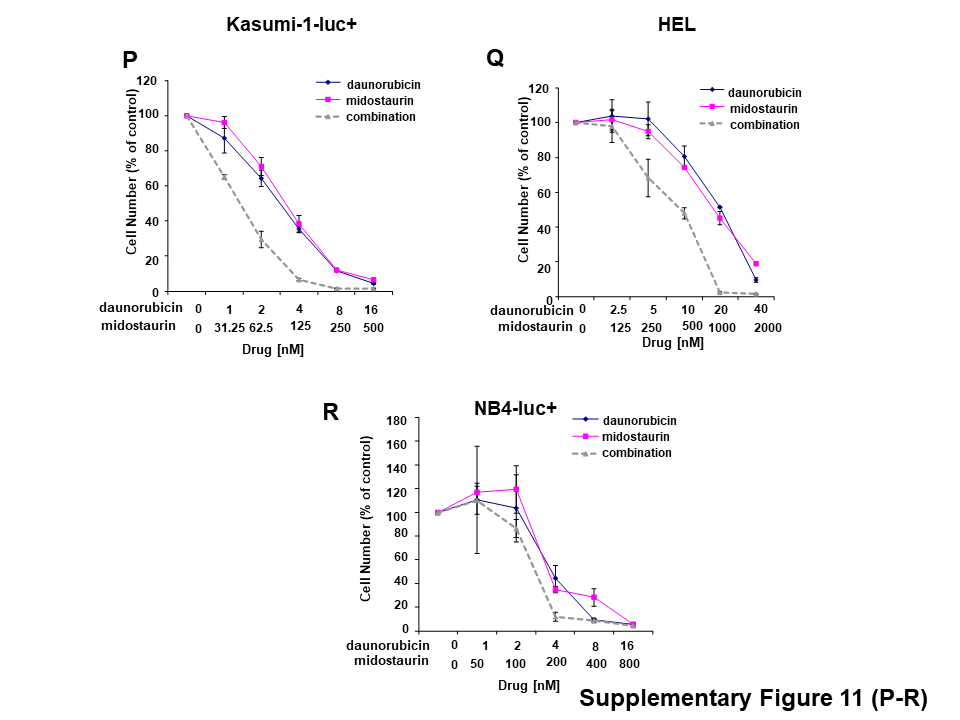

Supplement: Supplementary file 21 [file JCMM-24-2968-s021.TIF]

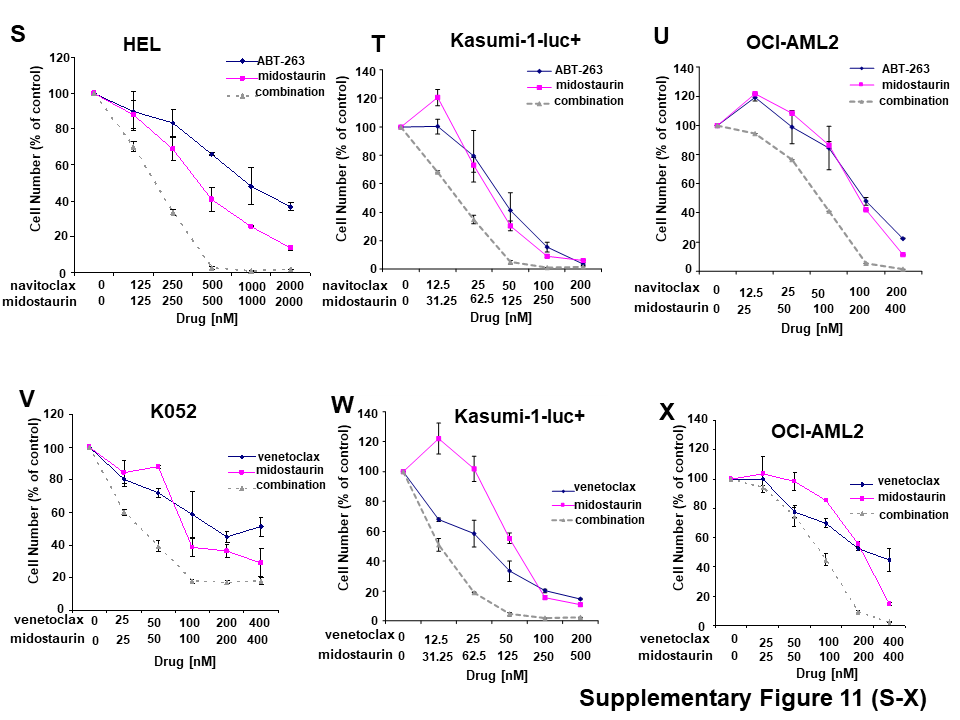

Supplement: Supplementary file 22 [file JCMM-24-2968-s022.TIF]

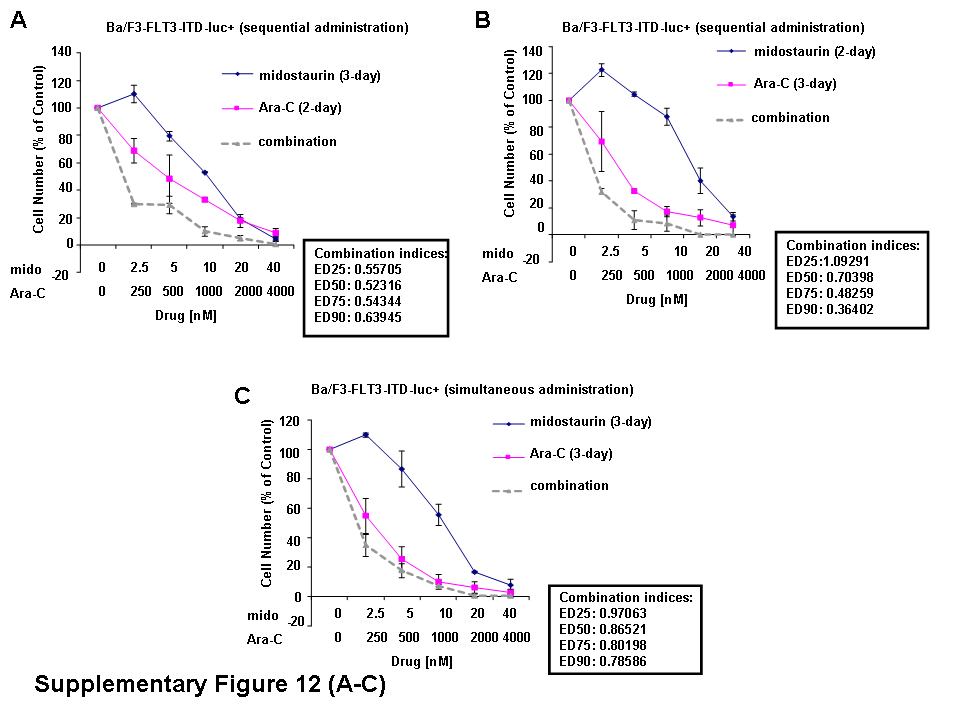

Supplement: Supplementary file 23 [file JCMM-24-2968-s023.TIF]

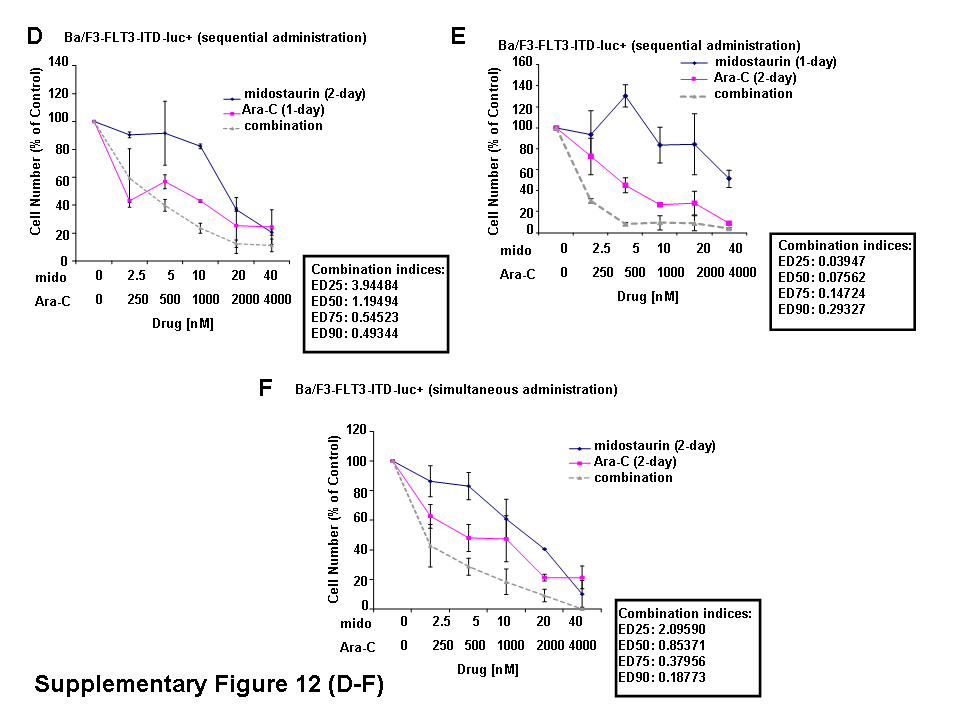

Supplement: Supplementary file 24 [file JCMM-24-2968-s024.TIF]

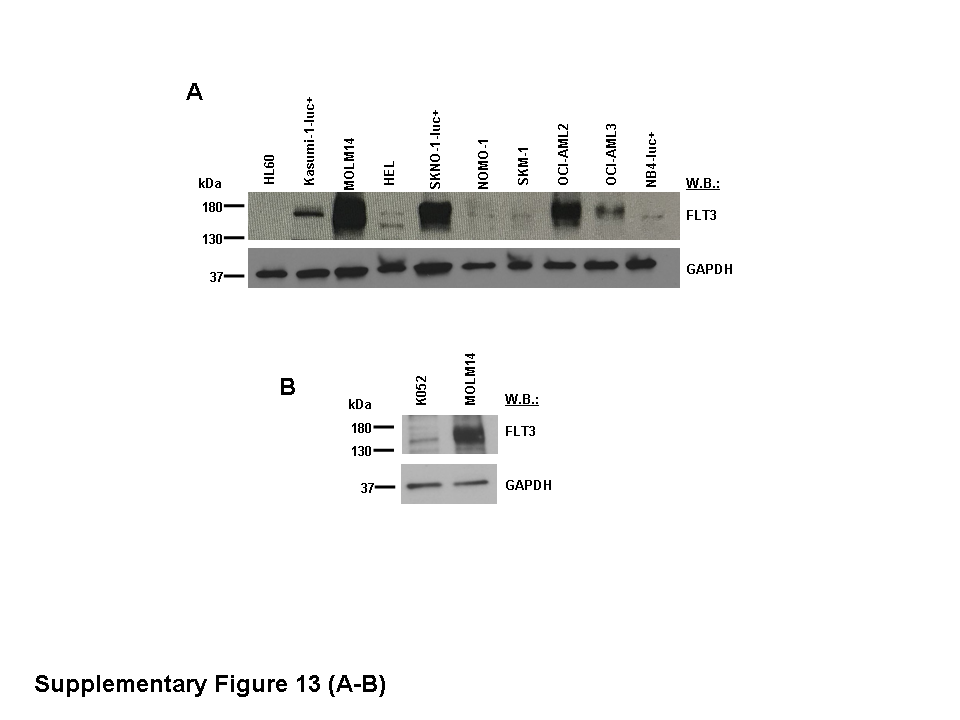

Supplement: Supplementary file 25 [file JCMM-24-2968-s025.TIF]

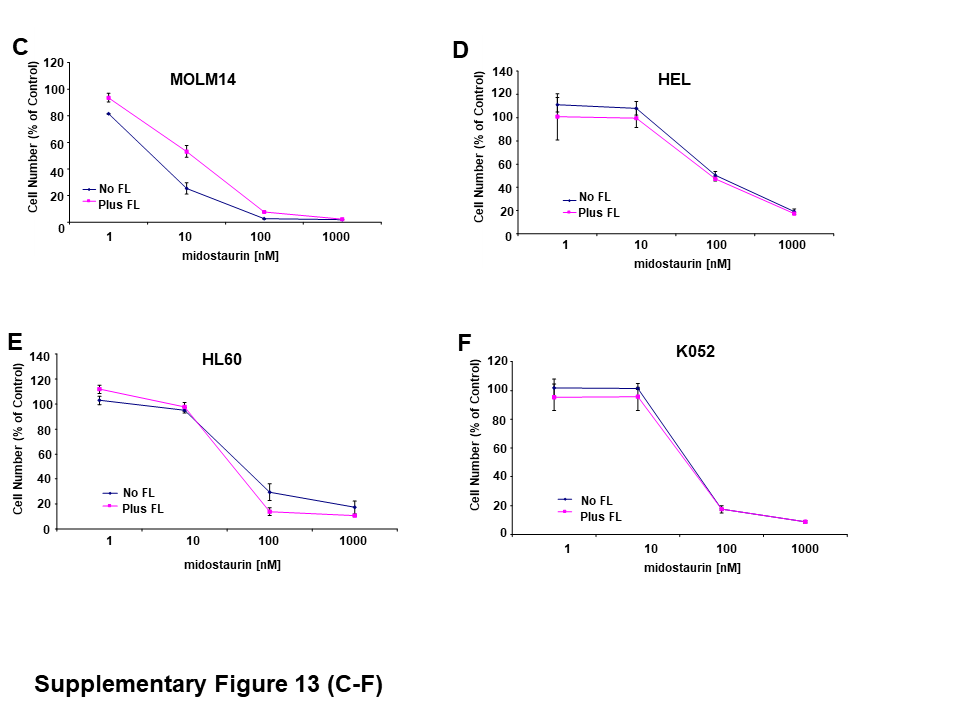

Supplement: Supplementary file 26 [file JCMM-24-2968-s026.TIF]

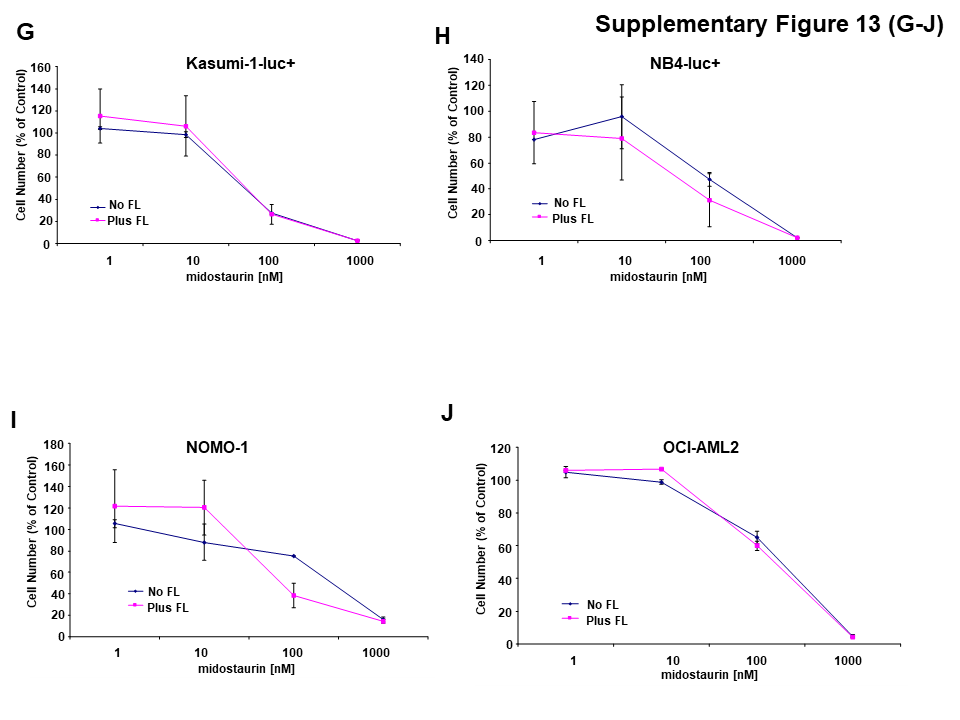

Supplement: Supplementary file 27 [file JCMM-24-2968-s027.TIF]

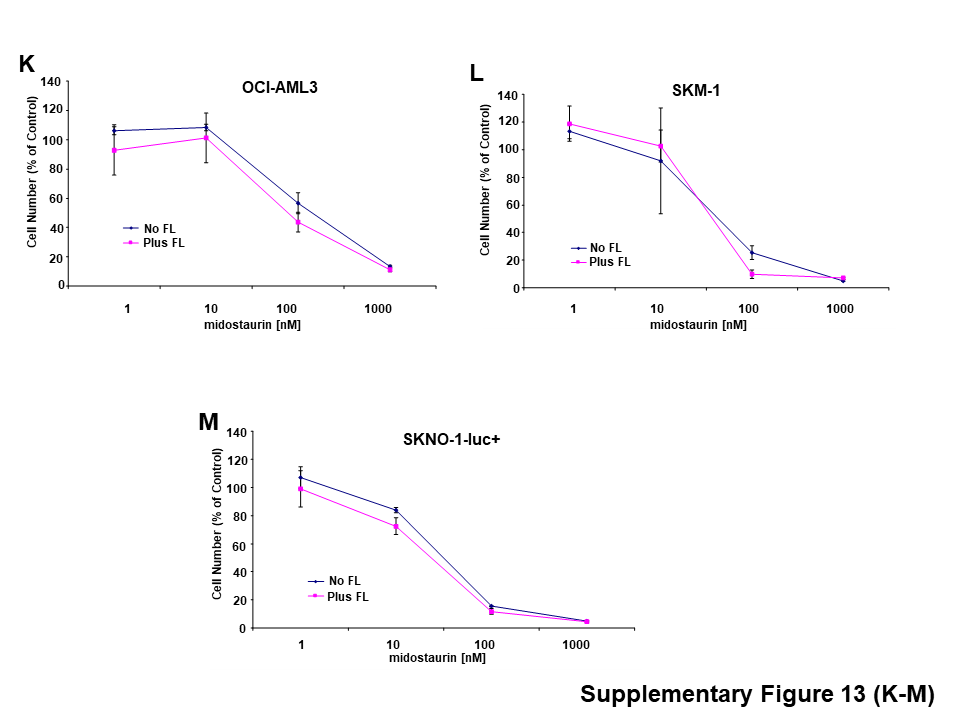

Supplement: Supplementary file 28 [file JCMM-24-2968-s028.TIF]
