# Supplementary material for: Effects of the multi‐kinase inhibitor midostaurin in combination with chemotherapy in models of acute myeloid leukaemia
Source: J Cell Mol Med. 2020 Jan 22;24(5):2968–80. doi: 10.1111/jcmm.14927 (PMC7077552; doi:10.1111/jcmm.14927)
Supplement: Supplementary file 29 [file JCMM-24-2968-s029.docx]

| **Patient**  **Sample** | **Gender**  **Age** | **Clinical diagnosis/Clinical history** | **Blasts** | **Cytogenetics and genetic mutations** |
| --- | --- | --- | --- | --- |
| **AML PT#2** | Male | Clinical diagnosis: AML in relapse  Clinical history: AML relapsed after transplant with refractory disease after 2 cycles on 14-222 MUC1+ decitabine | 95% (aspirate),  95%  (biopsy) | 45,XY,t(1;5)(q22;q33),t(3;4)(q27;q21),-7,inv(12)(p13q15),var(22)(p12)c[20].ish t(1;5)(PDGFRB-;PDGFRB+)[5]  Genetic mutations:  EZH2 p.R690H(37.5% of 432 reads)  PTPN11 p.N308D (28.5% of 695 reads)  RUNX1 p.N254fs (32% of 147 reads)  Read count analysis shows loss of IKZF1 and EGR (on 7p), loss of CUX1, LUC7L2, BRAF and EZH2 (on 7q), loss of ETV6 (on 12p).  FLT3-ITD is not detected. |
| **AML PT#3** | Male | AML | 99% | 47,XY,+13[3]/46,XY[16]/92,XXYY[2]  FLT3-ITD detected: Insertion of 12 nt 3' to nt 1826, followed by a duplication of nt 1788 - 1826 (total = 51 bp). The ITD is 7% of the total flt3 alleles in the specimen; this normally corresponds to 14% blasts. However, the specimen had 93% blasts on the day of this analysis. Apparently, there is a minor clone with a 51bp ITD. |
| **AML PT#5** | Male | Acute monoblastic leukemia, FAB M5a subtype | 90% | 47,XY,+8[3]/46,XY,-7,+8[17]  FLT3-ITD is not detected. |

**Supplementary Table I. Primary AML patient characteristics.**
