# Supplementary material for: Effects of the multi‐kinase inhibitor midostaurin in combination with chemotherapy in models of acute myeloid leukaemia
Source: J Cell Mol Med. 2020 Jan 22;24(5):2968–80. doi: 10.1111/jcmm.14927 (PMC7077552; doi:10.1111/jcmm.14927)
Supplement: Supplementary file 30 [file JCMM-24-2968-s030.docx]

**Supplementary Figure Legends**

**Supplementary Figure 1(A). Effects of midostaurin on signaling in mutant and wt FLT3-expressing AML.** (A) Effects of midostaurin on signaling molecules associated with apoptosis in Ba/F3-FLT3-ITD cells.

**Supplementary Figure 1 (B). Effects of midostaurin on signaling in mutant and wt FLT3-expressing AML**. Annexin/pi staining corresponding to data shown in Figure 2D. Kasumi-1-luc+ cells treated with midostaurin.

**Supplementary Figure 1 (C). Effects of midostaurin on signaling in mutant and wt FLT3-expressing AML.** Annexin/pi staining corresponding to data shown in Figure 2D. OCI-AML2 cells treated with midostaurin.

**Supplementary Figure 1(D-F). Effects of midostaurin on signaling in mutant and wt FLT3-expressing AML.** Effects of midostaurin at the indicated concentrations on phosphorylation of key molecules involved in PI3K/AKT and MAPK signaling in wt FLT3-expressing Kasumi-1-luc+ cells. Shown are total AKT, MAPK, and S6 protein levels.

**Supplementary Figure 1(G-H). Effects of midostaurin on signaling in mutant and wt FLT3-expressing AML.** (G) Effects of midostaurin at the indicated concentrations on phosphorylation of signaling molecules downstream of oncogenic FLT3 in MOLM14 cells. (H) Effects of midostaurin at the indicated concentrations on phosphorylation of key signaling molecules involved in PI3K/AKT and MAPK signaling in wt FLT3-expressing OCI-AML2 cells.

**Supplementary Figure 2. Murine models of wt FLT3 AML.** (A) Comparison of engraftment of Kasumi-1-luc+ cells and SKNO-1-luc+ cells: Mean total flux bioluminescence. (B) Measure of leukemia burden in vehicle- versus midostaurin-treated mice for OCI-AML3-luc+ xenograft model (corresponds to bioluminescence data shown in Figure 4D). (C-D) Mean body weights for SKNO-1-luc+ (C) and OCI-AML3-luc+ (D) xenograft models.

**Supplementary Figure 3. SKNO-1-luc+ and OCI-AML3-luc+ xenografts.** (A-C) SKNO-1-luc+ xenograft. (A) Supine and Prone (Low Scale). Day 1 Pre-treatment. Representative Images (n=5). (B) Supine and Prone (High Scale). Day 1 – 15. Representative Images (n=5). (C) Supine and Prone (High Scale). Day 43 – 57. Representative Images (n=5). (D) OCI-AML3-luc+ xenograft. Supine and Prone (Low Scale). Day 1 Pre-treatment. Representative Images (n=5).

**Supplementary Figure 4 (A-D). Comparison of effects of FLT3 inhibitors versus SYK inhibitors on growth of cells expressing activated SYK or mutant FLT3.** (A) Proliferation curves generated for Ba/F3-SYK-TEL cells treated for approximately three days with FLT3 inhibitors (crenolanib, quizartinib, midostaurin, and gilteritinib) or the targeted SYK inhibitor, PRT062607. (B,C) Growth inhibition curves generated for Ba/F3-SYK-TEL cells treated with targeted SYK inhibitors, PRT062607 and entospletinib. (D) Growth inhibition curves generated for Ba/F3-FLT3-ITD cells treated with targeted SYK inhibitors, PRT062607 and entospletinib.

**Supplementary Figure 4 (E-J). Comparison of effects of FLT3 inhibitors versus SYK inhibitors on growth of cells expressing activated SYK or mutant FLT3.** Ba/F3-SYK-TEL and Ba/F3-FLT3-ITD cells were treated for approximately three days in the absence of growth factor by midostaurin (E), gilteritinib (F), crenolanib (G), quizartinib (H), sorafenib (I), or PRT062607 (J).

**Supplementary Figure 5. Comparison of effects of FLT3 inhibitors against the growth of parental Ba/F3 cells and Ba/F3-SYK-TEL cells.** Cells were treated for approximately three days. PRT062607 was tested in parallel with the FLT3 inhibitors as a positive control. Parental Ba/F3 and Ba/F3-SYK-TEL cells were treated with midostaurin (A), gilteritinib (B), PRT062607 (C), crenolanib (D), quizartinib (E), and sorafenib (F).

**Supplementary Figure 6. Comparison of effects of FLT3 inhibitors against the growth of Ba/F3-SYK-TEL cells in the absence and presence of 20% WEHI-conditioned media (WEHI used as a source of IL-3).** Cells were treated for approximately three days. PRT062607 was tested in parallel with the FLT3 inhibitors as a positive control. Ba/F3-SYK-TEL cells were treated +/- IL-3 with midostaurin (A), gilteritinib (B), PRT062607 (C), crenolanib (D), quizartinib (E), and sorafenib (F).

**Supplementary Figure 7. Comparison of effects of FLT3 inhibitors against the growth of Ba/F3-FLT3-ITD cells in the absence and presence of 20% WEHI-conditioned media (WEHI used as a source of IL-3).** Cells were treated for approximately three days. Ba/F3-FLT3-ITD cells were treated +/-IL-3 with midostaurin (A), gilteritinib (B), PRT062607 (C), crenolanib (D), quizartinib (E), and sorafenib (F).

**Supplementary Figure 8. Effects of midostaurin alone and combined with standard chemotherapeutic agents against FLT3-ITD+ MOLM14 cells.** Cell lines were treated for approximately 3 days. Graphs shown are representative of two independent studies for which similar results were observed.

**Supplementary Figure 9. Effects of inhibitors of proviability signaling molecules on proliferation of human AML cell lines.** Treatment of AML lines with venetoclax (A), navitoclax (B), or S63845 (C). 3-day treatments. The venetoclax sensitivity of cell lines shown here is similar to that previously published (Pan R, Hogdal LJ, Benito JM, Bucci D, Han L,  Borthakur G, Cortes J, DeAngelo DJ, Debose L, Mu H,  Dohner H, Gaidzik VI, Galinsky I, Golfman LS, Haferlach T, Harutyunyan KG, Hu J,  Leverson JD, Marcucci G, Muschen M,  Newman R, Park E, Ruvolo PP, Ruvolo V, Ryan J, Schindela S,  Zweidler-McKay P, Stone RM, Kantarjian H, Andreeff M, Konopleva M, Letai AG. Selective BCL-2 inhibition by ABT-199 causes on-target cell death in acute myeloid leukemia. Cancer Discov. 2014 Mar;4(3):362-75).

**Supplementary Figure 10 (A-I). Effects of midostaurin alone and combined with 5-azacytidine against wt FLT3-expressing AML cell lines.** Cell lines were treated for approximately 3 days. Concentrations of 5-azacytidine were held steady and combined with a range of concentrations of midostaurin and tested against different wt FLT3-expressing AML cell lines.

**Supplementary Figure 10 (J-N). Effects of midostaurin alone and combined with decitabine against wt FLT3-expressing AML cell lines.** Cell lines were treated for approximately 3 days. Concentrations of decitabine were held steady and combined with a range of concentrations of midostaurin and tested against different wt FLT3-expressing AML cell lines.

**Supplementary Figure 11 (A-E). Effects of midostaurin alone and combined with 5-azacytidine against human wt FLT3-expressing AML cell lines.** Cell lines were treated for approximately 3 days.

**Supplementary Figure 11 (F-J). Effects of midostaurin alone and combined with decitabine against human wt FLT3-expressing AML cell lines.** Cell lines were treated for approximately 3 days.

**Supplementary Figure 11 (K-O). Effects of midostaurin alone and combined with Ara-C against human wt FLT3-expressing AML cell lines.** Cell lines were treated for approximately 3 days. Graphs shown are representative of two independent studies.

**Supplementary Figure 11 (P-R). Effects of midostaurin alone and combined with daunorubicin against human wt FLT3-expressing AML cell lines.** Cell lines were treated for approximately 3 days. Graphs shown are representative of two independent studies.

**Supplementary Figure 11 (S-X). Effects of midostaurin alone and combined with navitoclax or venetoclax against human wt FLT3-expressing AML cell lines.** Cell lines were treated for approximately 3 days. Graphs shown are representative of two independent studies.

**Supplementary Figure 12 (A-C). Sequential versus simultaneous administration of midostaurin and Ara-C against Ba/F3-FLT3-ITD-luc+ cells (3 day total study).** (A) Sequential administration of midostaurin (added first) and Ara-C (added second) against Ba/F3-FLT3-ITD-luc+ cells. (B) Sequential administration of midostaurin (added second) and Ara-C (added first) against Ba/F3-FLT3-ITD-luc+ cells. (C) Simultaneous administration of midostaurin and Ara-C against Ba/F3-FLT3-ITD-luc+ cells.

**Supplementary Figure 12 (D-F). Sequential versus simultaneous administration of midostaurin and Ara-C against Ba/F3-FLT3-ITD-luc+ cells (2-day total study).** (D) Sequential administration of midostaurin (added first) and Ara-C (added second) against Ba/F3-FLT3-ITD-luc+ cells. (E) Sequential administration of midostaurin (added second) and Ara-c (added first) against Ba/F3-FLT3-ITD-luc+ cells. (F) Simultaneous administration of midostaurin and Ara-C against Ba/F3-FLT3-ITD-luc+ cells.

**Supplementary Figure 13 (A-B). FLT3 expression levels in human AML lines.**

**Supplementary Figure 13 (C-F). Treatment of human AML cell lines for 3.5 days with midostaurin in the absence and presence of FLT3 ligand (FL).**

**Supplementary Figure 13 (G-J).** **Treatment of human AML cell lines for 3.5 days with midostaurin in the absence and presence of FLT3 ligand (FL).**

**Supplementary Figure 13 (K-M). Treatment of human AML cell lines for 3.5 days with midostaurin in the absence and presence of FLT3 ligand (FL).**
